# Characterization and *in vitro* Analysis of Probiotic-Derived Peptides Against Multi Drug Resistance Bacterial Infections

**DOI:** 10.3389/fmicb.2020.01963

**Published:** 2020-08-25

**Authors:** Aninda Mazumdar, Yazan Haddad, Vishma Pratap Sur, Vedran Milosavljevic, Sukanya Bhowmick, Hana Michalkova, Roman Guran, Radek Vesely, Amitava Moulick

**Affiliations:** ^1^Department of Chemistry and Biochemistry, Faculty of AgriSciences, Mendel University in Brno, Brno, Czechia; ^2^Central European Institute of Technology, Brno University of Technology, Brno, Czechia; ^3^Department of Traumatology at the Medical Faculty, Masaryk University and Trauma Hospital of Brno, Brno, Czechia

**Keywords:** antibacterial peptides, antibiotics, multidrug resistance, bacteria, infections

## Abstract

An inexorable switch from antibiotics has become a major desideratum to overcome antibiotic resistance. Bacteriocin from *Lactobacillus casei*, a cardinal probiotic was used to design novel antibacterial peptides named as Probiotic Bacteriocin Derived and Modified (PBDM) peptides (PBDM1: YKWFAHLIKGLC and PBDM2: YKWFRHLIKKLC). The loop-shaped 3D structure of peptides was characterized *in silico* via molecular dynamics simulation as well as biophysically via spectroscopic methods. Thereafter, *in vitro* results against multidrug resistant bacterial strains and hospital samples demonstrated the strong antimicrobial activity of PBDM peptides. Further, *in vivo* studies with PBDM peptides showed downright recovery of balb/c mice from Vancomycin Resistant *Staphylococcus aureus* (VRSA) infection to its healthy condition. Thereafter, *in vitro* study with human epithelial cells showed no significant cytotoxic effects with high biocompatibility and good hemocompatibility. In conclusion, PBDM peptides displayed significant antibacterial activity against certain drug resistant bacteria which cause infections in human beings. Future analysis are required to unveil its mechanism of action in order to execute it as an alternative to antibiotics.

## Introduction

Antibiotic resistant bacterial infections are a global health problem due to considerable threat to morbidity and mortality (Tacconelli et al., 2018; Bhowmick et al., 2020). The clinical need for the generation of new antibiotics is constantly putting pressure on pharmaceutical research and development. Whereas, antibiotic resistance is increasing due to incomplete course of antibiotic dose and its misuse (McNeece et al., 2014), exposure to constant stress, horizontal gene transfer and changes in genomic level (Ventola, 2015). The rapid increase in the number of resistant bacteria have forcefully reduced the use of antibiotics and an urge to introduce the development of new alternatives (Rincón et al., 2014). The World Health Organization (WHO) listed a report on Antibiotic Resistance in 2017, which classify resistance bacteria into three main categories depending on the priority of threat levels of bacterial pathogenicity like high, critical and medium (Tacconelli et al., 2018).

The *Staphylococcus aureus* and many of its relative taxonomic class are opportunistic pathogens of significant threat level being a common cause for hospital infections ranging from soft tissue infections to life threatening infections with some assistance in chronic infections (McGuinness et al., 2017). In the Intensive Care Unit (ICU) of many hospital, resistant bacterial strains like *Klebsiella pneumoniae* (9.7%), *Staphylococcus aureus* (10.7%), Enterococcus spp. (10.6%), *Stenotrophomonas maltophilia* (11.5%), *Pseudomonas aeruginosa* (15.6%), and *Acinetobacter baumannii* (19.5%) can be acquired which are responsible behind deadly infections that are difficult to treat (Tan et al., 2014). In burn patients cases, *S. aureus* can cause sepsis which leads to death (Thomer et al., 2016). The Vancomycin-resistant *Staphylococcus aureus* (VRSA) is even a greater threat due its ability to prevent vancomycin penetration into the cells, alterations in gene transcription and altered autolysis (Alexander et al., 2014). Thus, resistance toward vancomycin being the last resort against Gram-positive bacterial infections and other antibiotics have made VRSA a serious problem; also prioritized as the high threat level by WHO with high clinical global burden (Cui et al., 2006; Davies and Davies, 2010).

Antimicrobial peptides have started gaining interest due to their natural occurrence, permeating ability, providing defense against invading pathogens, acting as an element for innate immunity, amphipathic nature, disruption of cell wall and high effectivity (Zhu et al., 2006; Groh et al., 2015; Wang et al., 2017). They have limited bacterial resistance compared to the antibiotics. Currently, major limitations to the use of peptides include the expensive cost of production, and few information about their specificity (Brunetti et al., 2016). In order to address these limitations, we introduce our peptides of interest PBDM1 and PBDM2, derived from *Lactobacillus* sp. well-known probiotic bacteria. It had been reported *Lactobacillus* sp. when cultivated together with *S. aureus* showed inhibitory effects on *S. aureus* (Mohammedsaeed et al., 2014). PBDM1 and PBDM2 are short 12 amino acid sequence peptides derived from a bacteriocin present in *Lactobacillus casei.* The peptides are shorter version from m2163 and m2386 peptides reported previously (Tsai et al., 2015). Multiple sequence alignment of the four peptides is shown in Supplementary File Section 1 (Supplementary Figure S1). PBDM peptides are amphipathic peptides with positive net charge which was further studied to analyze its antibacterial activity (Lorenzón et al., 2012; Vicente et al., 2013).

Our main focus during the present study was to design, synthesize and characterize the PBDM peptides and examine their antimicrobial activity against multidrug resistant pathogenic strains. The results were promising against the antibiotic resistant bacteria with no toxicity and prominent hemocompatibility *in vitro* and high biocompatibility *in vivo*. Thus, PBDM peptides can be used for a better treatment strategy as a potent replacement for antibiotics with negligible toxicity and medicinal values.

## Materials and Methods

### Designing of PBDM Peptides

The peptide PBDM1 and PBDM2 were designed from a previously reported bacteriocin like peptide m2163 (KRKCPKTPFDNTPGAWFAHLILGC) present in *Lactobacillus casei* ATCC 334 (Tsai et al., 2015). Both the peptides were 12 amino acids long selected from the C-terminal sequence with the startup sequence as WFAHLILGC with no net charge. PDBM1 with the sequence YKWFAHLIKGLC was designed by adding Tyr and two Lys to increase the presence of aromatic amino acid, net positive charge of two and improved solubility. Whereas, in case of PDBM2 with sequence YKWFRHLIKKLC had similar modifications like PDBM1 with two additional replacements of an Ala to Arg and Gly to Lys, that increased the net positive charge to four, improves water solubility and stabilize the peptide backbone (Haug et al., 2016). It was previously reported that the Arg and Trp complement each other to increase antimicrobial activity (Chan et al., 2006). Finally, the choice of the C-terminus for cystination was to avoid too many modifications at both ends of peptide. Cys residue provides thiol (–SH) group which is capable of forming chemical bonds with other molecules (e.g., gold nanoparticles).

### Molecular Dynamics Simulation

GPU-accelerated Molecular Dynamics (MD) simulation was performed in isothermal-isobaric NPT ensemble (constant number, pressure, and temperature) using Gromacs v2018 on GeForce GTX 1080Ti card (Nvidia, Santa Clara, CA, United States). Peptide sequence of PBDM1 and PBDM2 (YKWFAHLIKGLC and YKWFRHLIKKLC) was used to build alpha helix structure in UCSF Chimera v1.10.2 (Backbone dihedrals Φ = −57° and Ψ = −47°; Side-chains via Dunbrack rotamer library). Positive charges were assumed for lysines and arginine while histidine protonation was assigned on Nε atom. The peptides were put in rhombic dodecahedron periodic box and solvated with 1920 and 1911 explicit SPC water molecules then neutralized with two and four Cl- ions in Amber99SB force field, respectively. Total number of atoms were 5976 and 5980 atoms. Box vector dimensions were 44.036 Å and 44.047 Å. Trajectories were calculated via leap-frog integrator every 2 fs. Neighbor searching was done via Verlet scheme while the Cut-off method was used for Van der Waals interactions at 12Å. For electrostatics calculations, the Particle Mesh Ewald (PME) was used with 12Å cutoff. The peptide was minimized via steepest descent (Convergence after 263 and 312 steps at maximum force < 1000 kJ/mol/nm, respectively). NVT ensembles (constant number, volume, and temperature) equilibration was done for duration of 1 ns using LINCS constraints for modified Berendsen thermostat coupling (300 K) in two groups (protein and non-protein), and H-bonds. Next, NPT ensemble equilibration for a duration of 1 ns with LINCS constraints for modified Berendsen thermostat coupling (300 K), H-bonds and isotropic Berendsen pressure coupling (1 bar). MD Production was performed in NPT ensemble for 1 microsecond using LINCS constraints for Parrinello-Rahman isotropic pressure coupling (1 bar), modified Berendsen thermostat coupling (300 K) and H-bonds. The total number of trajectory frames was 100,000 for which trajectories and energies were saved every 10 ps. Post MD analysis was done by putting the protein in the center of the box, followed by fitting of backbone and removal of all water molecules from the system. Standard DSSP method was used to perform time evolution of secondary structure. UCSF Chimera was used for visualization and further analysis. The most stable confirmations (largest clusters) was identified by clustering analysis of minimal backbone in steps of 50 frames. The representative 3D structures were superposed using Needleman-Wunsch alignment algorithm and BLOSUM-62 blocks substitution matrix. Root mean square deviations (RMSD) for all heavy atoms were calculated with reference to the representative frame of top cluster and to the first frame (α-helix).

### Chemicals, Synthesis and Characterization of the PBDM Peptides

All the chemicals for peptide synthesis, different assays and other chemicals were purchased from Sigma-Aldrich (St. Louis, MO, United States) in ACS purity, unless noted otherwise and to perform experiments and obtain the best results, sterile conditions were maintained.

PBDM1 (sequence – YKWFAHLIKGLC, with amidated C-terminus) and PBDM2 (sequence – YKWFRHLIKKLC, with amidated C-terminus) were synthesized using standard Fmoc solid phase synthesis on Liberty Blue peptide synthesizer (CEM, Matthews, NC, United States). Deblocking of Fmoc protecting group was performed with 20% piperidine v/v in *N,N*-dimethylformamide (DMF). Coupling was achieved using *N,N,N′,N′-*tetramethyl-O-(1H-benzotriazol-1-yl)uroniumhexafluorophosphate (HBTU), *N,N*-diisopropylethylamine (DIEA) and DMF. Cleavage of side chain protecting groups was performed by treating the peptides resin with 95% trifluoroacetic acid (TFA) *v/v*, 2.5% H_2_O *v/v* and 2.5% triisopropylpropylsilane v/v for 30 min at 38°C under microwave irradiation. Isolation of NVC was performed by multiple centrifugation (6000 rpm, 3 min) under cold diethyl ether then re-suspended in ACS water and lyophilized. Further, the formation of PBDM1–5(6)–Carboxyfluorescein and PBDM2–5(6)–Carboxyfluorescein conjugates were obtained using PBDM peptides and 5(6)–Carboxyfluorescein N–hydroxysuccinimide ester in the ratio of 10:1, kept in rotator Multi Bio RS–24 (Biosan, Riga, Latvia) for 24 h under constant rotation with time interval vibration. Thereafter, the HPLC (ESA Inc., Chelmsford, MA, United States) system consisted of two pumps ESA Model 584 and an autosampler ESA Model 542 (ESA Inc., Chelmsford, MA, United States) along with the column Kinetex EVO C18 (150 × 4.6 mm, 5 μm) was used to perform purification of the peptides and their conjugates. The wavelength was set to 214 nm and the injected sample volume was 20 μL. Mobile phase A and B consisted of water with 0.1% formic acid and methanol with 0.1% formic acid. Flow rate was 0.5 mL/min. Prior analyses the samples were diluted 100× with water containing 0.1% formic acid. The molecular weight of the peptide and its conjugate was verified by a MALDI-TOF mass spectrometer Bruker UltrafleXtreme (Bruker Daltonik GmbH, Germany). As a matrix 2,5-dihydroxybenzoic acid (DHB) prepared in 30% acetonitrile and 0.1% trifluoroacetic acid at concentration 20 mg/mL was used. The final spectrum was averaged from 5000 mass spectra per sample spot. Reflector positive mode was used. Laser power was set 5–10% above the threshold.

Finally, the ATR FT-IR spectra were collected using a Nicolet iS10 FT-IR spectrometer with a diamond attenuated total reflectance (ATR) attachment (Thermo Electron Inc., San Jose, CA, United States). Initially, to the diamond crystal of the ATR cell, the sample was added drop-wise (5 μL) and then the film was measured after spontaneous evaporation of the solvent. At a resolution of 4 cm^–1^, the IR spectra were recorded from 4000 to 650 cm^–1^ at 22°C. Each spectrum was acquired by adding together 64 interferograms. Lastly, the fluorescence emission and absorbance spectra of PBDM peptides and their conjugates were obtained using a multifunctional micro-titration plate reader, Tecan infinite M200 PRO (Tecan group Ltd., Mannedorf, Switzerland) (Moulick et al., 2018). The absorbance spectrum was measured within the range from 230 to 850 nm with the 2 nm step. For the fluorescence spectra measurement, 230 nm was used as excitation wavelengths and the fluorescence scan was measured within the range from 260 to 650 nm with the 2 nm step. The fractions were placed in UV-transparent 96 well microplate with flat bottom by Costar^®^ (Corning Inc., Corning, NY, United States).

### Cultivation of Bacterial Strains

Bacterial strains (*Staphylococcus aureus* NCTC 8511, Methicillin-Resistant *Staphylococcus aureus* (MRSA) ST239: SCCmecIIIA CCM 7111, Vancomycin-Resistant *Staphylococcus aureus* (VRSA) CCM 1767, Vancomycin-Resistant *Enterococcus faecalis* (VRE) ATCC 51299, *Escherichia coli* ATCC BAA 2340, *Enterococcus faecalis* ATCC 11700) were obtained from the National Collection of Type Cultures, England; American Type Culture Collection (ATCC), United States and the Czech Collection of Microorganisms, Faculty of Science, Masaryk University, Brno, Czechia. To the Mueller Hinton (MH) media of 15 mL in Erlenmeyer flask bacteria was inoculated and kept at 37°C, 130 rpm for 24 h. Further, the cultures were diluted using the Mueller Hinton (MH) broth to 0.1 Absorbance [0.5 MacFarland (McF) standards] at OD_600__nm_ and used for successive experiments (Jelinkova et al., 2018a).

### Antibacterial Assays

#### Growth Curves, Minimum Inhibitory Concentration (MIC) Determination and Viability Percentage

To determine the susceptibility of bacterial cultures the standard broth micro-dilution method (European Committee on Antimicrobial Susceptibility Testing) was used and detection was done by an unaided eye. The PBDM peptides were incubated with bacterial cultures (OD_600__nm_ = 0.5 McF and final dilution 1:100 with MH medium) in different concentrations (50, 25, 20, 10, and 5 μg/mL) at 37°C for 24 h. The lowest inhibiting concentration of the antimicrobial agent against each bacterium is the MIC of the antimicrobial agent with respect to that bacterium (Mazumdar et al., 2020).

The antibacterial activity of the PBDM peptides were determined using the Multiskan EX (Thermo Fisher Scientific, Waltham, MA, United States) by measuring the absorbance to obtain the growth curves. Different concentrations (50, 25, 20, 10, and 5 μg/mL) of the peptides were used to check the antibacterial activities. The control was without the peptides. Microplates with the volume of 300 μL were used to measure the absorbance. The solutions with different concentration of antimicrobial agent were added to the microplate wells and mixed with the bacterial cultures (0.5 MacFarland with final dilution 1:100 using MH medium). Later the plate was used in Multiskan EX for 24 h and results were evaluated the next day after 24 h to obtain the growth curves (Jelinkova et al., 2018a; Mazumdar et al., 2020; Sur et al., 2020a).

Further, the viability percentage was also calculated by using Multiskan EX, the final absorbance value at OD_600__nm_ after 24 h of treatment of bacteria using the different concentrations (50, 25, 20, 10, and 5 μg/mL) of peptides in comparison with the positive control (Jelinkova et al., 2018b; Mazumdar et al., 2020).

#### Colony Forming Unit (CFU) Assay

The bacterial strains were grown in MH medium for overnight at 37°C in a shaking incubator at 140 rpm. The very next day the culture was diluted to OD_600__nm_ = 0.1, in fresh MH medium overnight in the same condition until the OD_600__nm_ = 0.3–0.5 was obtained. The culture was then diluted to OD_600__nm_ = 0.1 and further diluted by 1:100 dilution factor which was followed by adding the peptides at their pre–determined Minimum Inhibitory Concentrations (MIC) (as mentioned in Table 2). Thereafter, the samples were drawn and results were obtained after 24 h incubation. Dilutions were made and the culture was spread on MH agar plates, which was incubated at 37°C incubator overnight and CFUs were determined the next day. Bacterial culture without PBDM peptides treatment wasere used as negative control and positive control was spread of untreated bacterial culture on agar plates prepared with tetracycline (10 μg/mL) and cefoxitin (32 μg/mL)kanamyicin (Zhou et al., 2012; Haque et al., 2017; Sur et al., 2019).

### Application of Peptide on Bacterial Samples From Hospital Patients

The collection of swabs from infected wounds of three patients with the proper signed information and consent of the volunteer and subsequent bacteria cultivation was carried out according to Hegerova et al. (2017). Patient’s enrollment into the clinical study was approved by the Ethics Committee of Trauma hospital in Brno, Czechia in accordance to act no. 378/2007 coll. For the bacteria identification, bacterial DNA was extracted using the Nucleo Spin Microbial DNA kit (Macherey-Nagel, Duren, Germany). The samples were obtained from the three different hospital patients and named accordingly (P1, P2, and P3). To amplify the 16S rRNA gene fragments, primers 27F-CC and 1492R were employed (Frank et al., 2008). Amplified products were purified analyzed using Sanger sequencing platform and obtained sequences were queried against the standard non-redundant nucleotide database (nr/nt) using Basic Local Alignment Search Tool for nucleotides (BLAST, blastn suite^1^). Multiple sequence alignment was performed using Clustal omega (Link^2^) to obtain the phylogenetic tree as shown in Supplementary Section 3. Later, the isolated samples were inoculated in MH broth and kept overnight at 37°C. They were diluted to obtain 0.1 Absorbance or 0.5 MacFarland with final dilution of 1:100 to study the growth curve in presence of different concentrations (50, 25, 20, 15, and 10 μg/mL) of PBDM peptides. Control was the sample bacteria without any treatment.

### Microscopy

#### Microscopy of PBDM Against Bacteria in Ambient Light and Live/Dead Cell Assay and Detection of VRSA Using PBDM Peptides Conjugate

Initially the samples were incubated with bacteria culture and PBDM peptides (respective MIC) at 37°C for 4 h in shaking incubator. To study the antibacterial activity of the peptides against *S. aureus*, MRSA, VRSA, *E. faecalis*, VRE, and *E. coli* using respective MIC values of PBDM peptides (as mentioned in Table 2), the optical Olympus BX51 fluorescence microscope equipped with a 40X phase contrast lens was used. The number of cells visualized per samples was observed from 10 randomized microscopic grid fields.

Thereafter, an inverted Olympus IX 71S8F-3 fluorescence microscope (Olympus Corporation, Tokyo, Japan) equipped with Olympus UIS2 series objective LUCPlanFLN 40 × (N.A. 0.6, WD 2.7–4 mm, F.N. 22), a mercury arc lamp X-cite 12 (120 W; Lumen Dynamics, Mississauga, ON, Canada), and, a Camera Olympus DP73 was used for live/dead cell imaging and bright field microscopy. The images were processed using the Stream Basic 1.7 Software. The bacterial samples were incubated with respective MIC values of PBDM peptides for 4 h at 37°C in a shaking incubator. The two fluorescent dyes, SYTO9 stain cells by permeating both damaged and intact cell membranes and propidium iodide (PI) to stain the cells with damaged cell membranes (Jelinkova et al., 2018a). The optical bright field microscopic (Boulos et al., 1999) image analysis was also performed for both peptides with respective MIC values of PBDM peptides against *S. aureus*, MRSA, VRSA, *E. faecalis*, VRE, and *E. coli*, with number of cells visualized per samples was observed from 10 randomized microscopic grid fields. Furthermore, VRSA samples were incubated with PBDM peptide conjugates for 4 h at 37°C in a shaking incubator and observed under microscope (Milosavljevic et al., 2016; Hussain et al., 2018; Mazumdar et al., 2020). The number of cells visualized per samples was observed from 10 randomized microscopic grid fields.

#### Detection of VRSA by PBDM–5(6)–Carboxyfluorescein and Cryo-SEM (Scanning Electron Microscope) Image Analysis to Understand the Mechanism

The VRSA cells were incubated with PBDM–5(6)–Carboxyfluorescein conjugates (15 and 10 μg/mL for PBDM1–5(6)–Carboxyfluorescein and PBDM2–5(6)–Carboxyfluorescein, respectively) for 30 min in dark and visualized under an inverted Olympus IX 71S8F-3 fluorescence microscope as discussed in the previous section. The images were processed using Stream Basic 1.7 Software (Milosavljevic et al., 2016).

Whereas, for Cryo-SEM the VRSA incubated for 4 h with PBDM peptides (15 and 10 μg/mL for PBDM1 and PBDM2, respectively) at 37°C in a shaking incubator and the control was VRSA without any treatment. Then the Cryo-SEM experiment method of plunge freezing was used. For plunging and storing of samples liquid nitrogen was used. Cryo-SEM visualization of samples was performed with FEI Versa3D equipped with a Quorum Cryo stage and transfer station (FEI Company) (Wu et al., 2014).

### Testing of the Cytotoxicity (MTT Assay) and Estimation of Hemocompatibility Against Eukaryotic Cells

The HBL 100 (mammary gland epithelial cells) and MDA MB 468 (mammary gland adenocarcinoma cells) human cell lines were cultured by immortalization of cells in RPMI-1640 medium with 10% fetal bovine serum, supplemented with penicillin (100 U/mL) and streptomycin (0.1 mg/mL). The treatment with different concentrations of PBDM peptides (125, 62.5, 31.25, 15.63, 7.81, 3.9, 1.95, 0.98, 0.49, 0.24, and 0.12 μg/mL) was initiated after the cells reached ∼60 – 80% confluence. They were used to study the viability using MTT [3-(4,5-dimethylthiazol-2-yl)-2,5-diphenyltetrazolium bromide] assay. Briefly, the suspension of 5000 cells in 50 μL medium was added to each well of microplates, followed by incubation for 24 h at 37°C with 5% CO_2_. After 24 h treatment using PBDM peptides, 10 μL of MTT [5 mg/mL in phosphate buffered saline (PBS)] was added to the cells and incubated for 4 h at 37°C. After that, MTT-containing medium was replaced by 100 μL of 99.9% dimethyl sulfoxide (DMSO) for 5 min incubation, absorbance of the samples was determined at 570 nm using Infinite m200 PRO (Tecan, Männedorf, Switzerland) (Heger et al., 2016). Further, the IC_50_ value of the PBDM peptides were calculated (Supplementary Section S10).

For hemocompatibility, Red blood cells (RBCs) were diluted with PBS (pH 7.4) and subsequently PBDM were added to RBCs solution in different concentration separately (40, 20, and 10 μg/mL) incubate at 37°C for 1 h. The positive and the negative control was 0.1% Triton X-100 and PBS, respectively. Thereafter, the samples were centrifuged at 3000 × *g* for 10 min and the absorbance of the samples was measured at 540 nm (Jelinkova et al., 2018a; Mazumdar et al., 2020). The formula is provided in the Supplementary Section 5.

### *In vivo* Study of VRSA on BALB/C and the Treatment Using PBDM Peptides

The preparation of *in vivo* model infection and treatment was performed using the 7–8 weeks old, 18 to 19.5 g weight female Balb/c mice. Mice were divided into 4 sets each contains 3 balb/c mice (Table 1). They were anesthetized using an intramuscular injection of a mixture of xylazine (Rometar,^®^ Spofaa.s., Prague, Czechia) at 10 mg/kg and ketamine (Narkamon,^®^ Spofaa.s., Prague, Czechia) at 100 mg/kg with an 1 mL insulin syringe (BD Veo™ insulin syringes with BD Ultra-Fine™ 6 mm × 31G needle) (Welberg et al., 2006; Gargiulo et al., 2012; Spunda et al., 2018; Sur et al., 2020b). The fur was removed from mice using Nair^®^ hair removal solution (Church & Dwight Co., Inc., Princeton, NJ, United States) and electric trimmer, 1 day prior to the experiment (Malachowa et al., 2013). The experimental condition throughout were maintained at 22 ± 1°C, light administration (12 h L and 12 h D) with maximum illumination of 200 lux and 60% humidity. The negative control was balb/c mice with no treatment and the positive control were balb/c mice infected with VRSA without any treatment. The experiments were approved by the Ethics Commission at the Faculty of AgriSciences, Mendel University in Brno, Czechia in accordance with Act No. 246/1992 Coll. to protect the animal from cruelty.

**TABLE 1:** Preparation of animal sets.

*Animal Set*	*Purpose of use*
*One*	Uninfected control
*Two*	Infected control without treatment
*Three*	Infected mice treated with PBDM1
*Four*	Infected mice treated with PBDM2

#### Introducing the Skin Infection

An overnight VRSA culture was cultivated at 37°C before the administration of the infection. The absorbance at 600 nm was used to make the culture 0.1 absorbance. Skin infection was introduced by subcutaneous injection with 0.05 mL of inoculum of VRSA (concentration 10^7^ CFU/mL) using 1 mL insulin syringe (Malachowa et al., 2013; Hussain et al., 2018; Sur et al., 2020b).

#### Treatment With PBDM Peptides and Monitoring the Mice

The treatment of the VRSA infected mice was done two times a day with PBDM1 and PBDM2 peptides, respectively, by subcutaneous injection at the site of infection and topical administration of the peptides on the surface of the exposed infected area until the mice recovered. During the treatment the final concentration of PBDM1 and PBDM2 peptides final concentration was 15 and 10 μg/mL, respectively. The images were taken using digital camera (Nikon digital camera (D40), NIKON Corp., Japan) to keep the record of the changes that underwent before and after the treatment (Koch, 2006; O’Toole et al., 2012; Sevgi et al., 2013; Sykes et al., 2014; Sur et al., 2020b). The images of the control set were also take using the same camera.

## Results and Discussion

The design, synthesis and characterization of the PBDM peptides were done using different computational, biophysical and biochemical technique. Thereafter, antibacterial activity of the peptides, against *S. aureus*, VRSA, MRSA, VRE, *E. coli*, *E. faecalis* and hospital samples from live patients were studied. Followed by the understanding of the mechanism of action using Cryo-SEM. Further, the *in vitro* and *in vivo* studies helped to obtain the information about peptide toxicity, biocompatibility and medicinal value. Since, VRSA has a high resistance toward almost all antibiotics effective against gram positive bacteria, thus it proves to be an ideal test model for a novel antimicrobial agent (Jelinkova et al., 2018a). Thus, VRSA infection was introduced in balb/c to which PBDM peptides were applied and change in infection was studied.

### Molecular Dynamics Simulation Analysis

**FIGURE 1:**
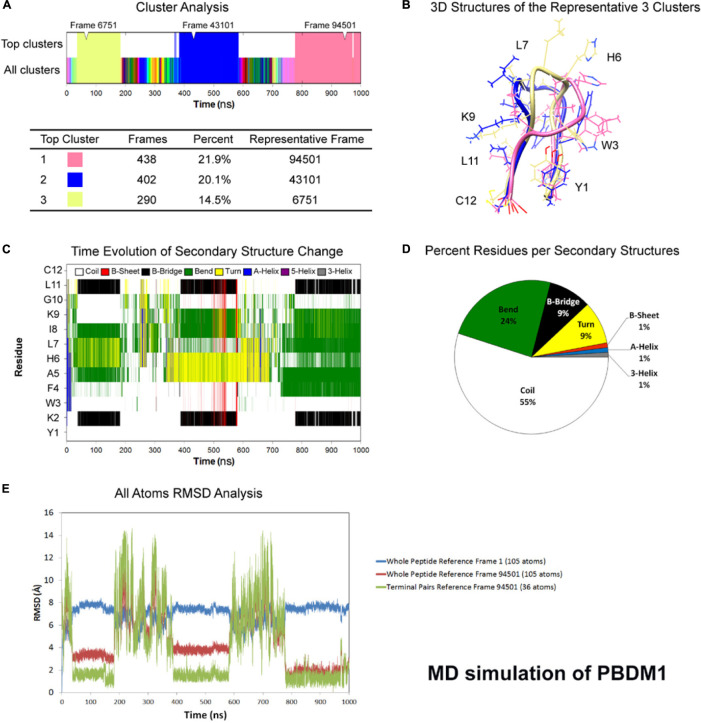
Atomistic molecular dynamics of PBDM1 in explicit water in NPT ensemble for 1000 ns at 300 K temperature. **(A)** Cluster analysis showing three major clusters covering 56.5% of simulation time (∼565 ns). **(B)** 3D structures of the representative frames of the three major clusters. The terminal pairs YK (N-terminus) and LC (C-terminus) have very stable conformations and very low deviation below 2Å. **(C)** Time evolution graph of secondary structure change with clear formation of B-Bridge between K2 and L11 (black color). The bends and turns are clear in the center of the peptide (green and yellow colors, respectively). **(D)** Secondary structures described by percent residues per assignment, and calculated by averaging the counts of residues for each secondary structure assignment in each frame. Coils, Bends, B-bridges and turns are predominant. **(E)** Root mean square deviations (RMSD) analysis. In the timeframe of the three major clusters, whole peptide RMSD against first frame (alpha-helix) was in the range of 6–8 Å. Whole peptide RMSD against the major cluster was in the range 4 Å or lower.

**FIGURE 2:**
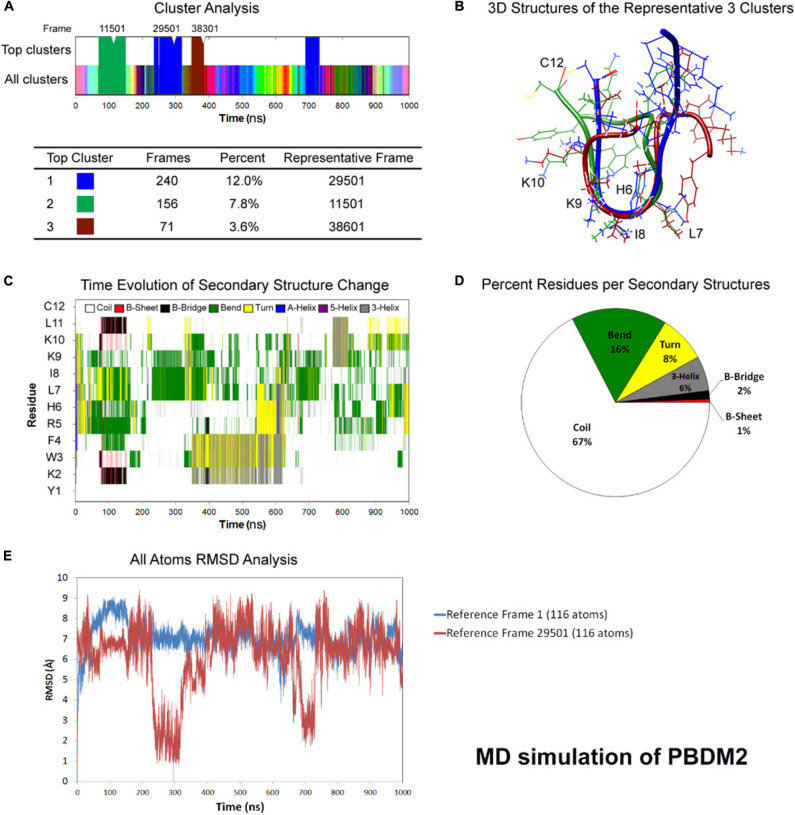
Atomistic molecular dynamics of PBDM2 in explicit water in NPT ensemble for 1000 ns at 300 K temperature. **(A)** Cluster analysis showing top three major clusters covering 23.4% of simulation time (∼234 ns). **(B)** 3D structures of the representative frames of the three major clusters. The most similar conformation is in the center of the peptide between sixth and ninth residues. **(C)** Time evolution graph of secondary structure change. **(D)** Secondary structures described by percent residues per assignment, and calculated by averaging the counts of residues for each secondary structure assignment in each frame. Coils were the most predominant. **(E)** Root mean square deviations (RMSD) analysis shows deviations in the range of 5–9 Å. The top cluster showed RMSD values below 4 Å.

One microsecond MD simulation was used to study the structure of the peptides at room temperature condition. For PBDM1 and PBDM2 peptides, the average total energy of simulation was −6.59e + 04 kJ/mol (−8.09e + 04 kJ/mol potential energy and 1.50e + 04 kJ/mol kinetic energy) and −6.69e + 04 kJ/mol (−8.20e + 04 kJ/mol potential energy and 1.50e + 04 kJ/mol kinetic energy), respectively. Average temperature was 3.00e + 02 K for both the peptide and average pressure was 1.79 bar and 1.45 bar. Cluster analysis of trajectories every 500 ps showed three stable and highly similar clusters (Figures 1A,B) but in case of PBDM2 every 500 ps showed the top three major clusters covering 23.4% of simulation time (Figure 2A), which is indicative of the random conformations throughout the simulation. The PBDM1 The PBDM1 peptide forms a U-shaped backbone with highly stable termini for periods of ∼200 ns (Figures 1A,B). Upon detachment of the B-bridge between K2 and L11 residues, the peptide switches to random coil secondary structure for ∼200 ns as well (Figure 1C). Average secondary structure compositions were Coil (55%), Bend (24%), B-bridge (9%), and Turn (9%) (Figure 1D). RMSD analysis showed similarity in the three major clusters as compared to the random coil conformation (Figure 1E). The whole peptide RMSD against first frame (alpha-helix) was in the range of 6–8 Å. Whole peptide RMSD against the representative frame of the major cluster was in the range 4 Å or lower. The terminal pairs YK (N-terminus) and LC (C-terminus) have very stable conformations and very low deviation below 2 Å.

Whereas in case of PBDM2 peptide, most of the conformational similarity between the three top clusters was in the HLIK loop (sixth to ninth residues) (Figure 2B). A B-Bridge between K2 and L11 residues was formed within the first 100 ns of simulation, however, it was not stable due to possible positive charge repulsion between K2 and K9/K10 residues (Figure 2C). Average secondary structure compositions were Coil (67%), Bend (16%), Turn (8%), and 3-Helix (6%) (Figure 2D). RMSD analysis shows deviations in the range of 5–9 Å. The top cluster showed RMSD values below 4 Å (Figure 2E). Despite the peptide sequence of alternating residues (two charged and two hydrophobic), no alpha helix was observed. On the other hand, the stability of a U-shaped conformation was destabilized by the repulsion between the positively charged lysines.

Molecular dynamics simulation showed the PBDM1 peptide to alternate between stable U-shaped backbone and random coil in 200 ns intervals. One explanation for the stability of the U-shaped conformation is the stability of the terminal pairs YK (N-terminus) and LC (C-terminus). Also the proximity of aromatic side-chain of F4 residue to aliphatic side-chain of L11 and the carbon chain of K2 can play role via hydrophobic interactions. But in case of PBDM2 peptides the structure was mainly random coil and U-shaped conformation was destabilized due to the presence of positively charged lysines and the repulsion between them.

### Characterization of PBDM

The PBDM peptides and their conjugates were purified using HPLC-UV. The chromatogram for PBDM1 contains two peaks showing at two different retention time. The peak 1 denotes PBDM1 and peak 2 is the PBDM1–5(6)–Carboxyfluorescein conjugate as shown in Supplementary Figure S11A. Whereas in case of PBDM2 similar results were seen but the retention time were different as shown in Supplementary Figure S11B. The eluates obtained from the HPLC-UV at respective time intervals were used to perform MALDI-TOF mass spectrometry.

The MALDI-TOF MS for each samples were performed and two different peaks were obtained which represents PBDM peptides and their related PBDM–5(6)–Carboxyfluorescein conjugates as shown in Supplementary Figure S11D and S11F. The peak of PBDM1 and PBDM2 peptides were obtained at 1477.605 Da and 1633.797 Da whereas, their conjugates produced peaks at 1835.632 Da and 1991.803 Da, respectively. The mass difference of 358.03 and 358.01 were seen due to the displacement of N–hydroxysuccinimide (115.09 Da) after formation of the PBDM–5(6)–Carboxyfluorescein. Thus, the formation of conjugates was confirmed.

The absorbance spectra were obtained for PBDM–5(6)–Carboxyfluorescein conjugates, with each showing similar pattern in spectrum (305 to 1000 nm) with absorbance maxima at 485 and 470 nm, respectively but PBDM peptides showed no prominent absorbance. Thus the formation of conjugate showed visible change in absorbance spectra as shown in Supplementary Figure S11C. The reported λ_em_ (emission) of Carboxyfluorescein N-hydroxysuccinimide ester is at 518 nm (Keller et al., 2002). Similarly, the emission spectra (380 to 600 nm) for the PBDM1 and PBDM2 conjugates showed the λ_max_ (emission maxima) peak at 540 and 542 nm with high fluorescence intensity but no emission was observed for PBDM peptide (Supplementary Figure S11E). Thus, the conjugates can be used for detection of bacteria using fluorescence microscope in the red fluorescence region.

ATR-FT-IR analysis (Supplementary Figure S11G) of the amide I band correlated with MD simulation results. The amide I (peak at 1650 cm^–1^) corresponds to the *C* = O stretching and it is clear indication of vibrations that are connected with alpha helix and random coil. Furthermore, the peak indicates absence of beta-sheet, a signature of short range interaction between backbones seen in peptide aggregations (Adochitei and Drochioiu, 2011). The amide II band (peak at 1539 cm^–1^) and amide III band (double peak at 1200–1210 cm^–1^) correspond to N-H bending and C-N stretching. The broad bands in 2500–3500 cm^–1^ correspond to O-H (peak at 3290–3305 cm^–1^) and C-H (double peak at 2945–2965 cm^–1^) stretching (Coates, 2006).

Finally, after the characterization of the PBDM peptides, they were used to study its antibacterial efficacy using different microbiological assays. Whereas the PBDM–5(6)–Carboxyfluorescein conjugates were used for the detection of the interaction of bacteria with the peptides using fluorescence microscope.

### PBDM Activity Against Different Bacterial Strains

The initiation to understand the antibacterial efficacy of PBDM peptides was done by determining their MIC (shown in Table 2) against *S. aureus*, MRSA, VRSA, *E. faecalis*, VRE and *E. coli* using broth microdilution method. The MIC value of PBDM1 was found to be as 10 μg/mL against *S. aureus*, MRSA, and *E. coli*. Whereas, in case of *E. faecalis*, VRSA and VRE the MIC was found to be 30, 15, and 20 μg/mL. But, the MIC value for PBDM2 was 10 μg/mL when tested against *S. aureus*, MRSA, VRSA, VRE, and *E. coli*. Whereas, in case of *E. faecalis* the MIC was found to be 20 μg/mL. The concentration below the MIC value showed turbid solution which concludes the presence of bacteria with less or no antibacterial activity (Mazumdar et al., 2020).

**TABLE 2:** Minimum Inhibitory Concentrations (MICs) of PBDM1 and PBDM2 by broth micro–dilution method.

Bacterial Strains	PBDM1 (μg/mL)	PBDM2 (μg/mL)
*S. aureus*	10	10
MRSA	10	10
VRSA	15	10
*E. faecalis*	30	20
VRE	20	10
*E. coli*	10	10

**FIGURE 3:**
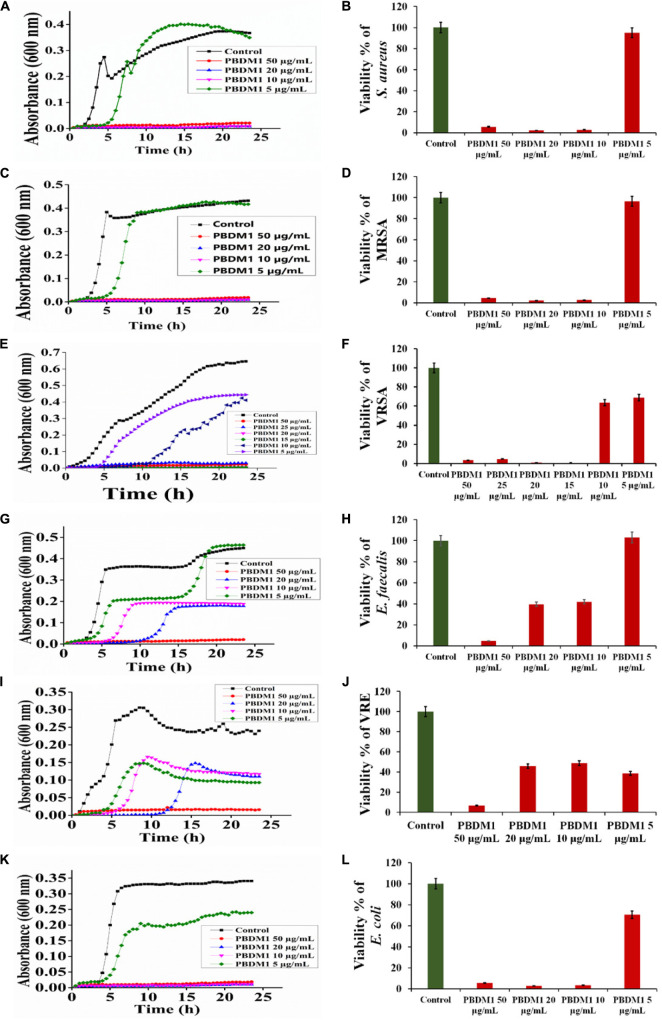
The Growth curve and Viability percentage of *S. aureus* **(A,B)**, MRSA **(C,D)**, VRSA **(E,F)**, *E. faecalis* **(G,H)**, VRE **(I,J)** and *E. coli* **(K,L)** in presence of PBDM1. Data represent the mean ± SD, *n* = 3.

**FIGURE 4:**
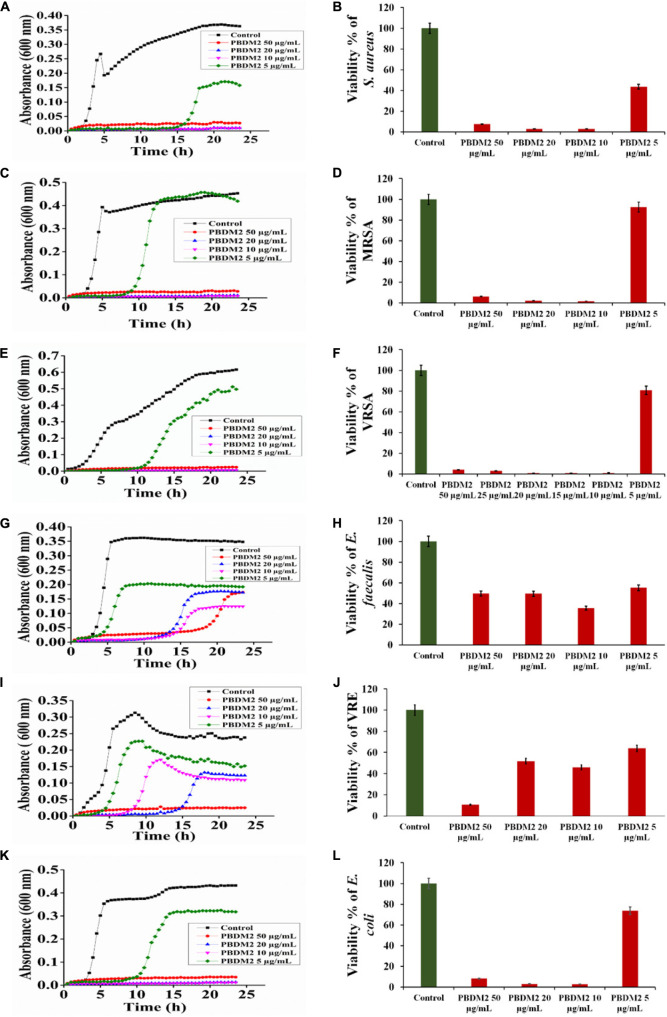
The Growth curve and Viability percentage of *S. aureus* **(A,B)**, MRSA **(C,D)**, VRSA **(E,F)**, *E. faecalis* **(G,H)**, VRE **(I,J)** and *E. coli* **(K,L)** in presence of PBDM2. Data represent the mean ± SD, *n* = 3.

Thereafter, the growth curve analysis was performed with *S. aureus*, MRSA, VRSA, *E. faecalis*, VRE, and *E. coli* in the presence of PDBM peptides to study the growth pattern of each bacterium over 24 h time duration. The PBDM1 treated *S. aureus* showed high inhibition of 94.3% at 50 μg/mL and lower in concentration of 20 and 10 μg/mL also showed inhibitions of more than 94% but further lower concentration of 5 μg/mL showed no inhibition (Figure 3A). In case of PBDM2 treated *S. aureus* steady 93% inhibition at 50 μg/mL and similar effects for 20 and 10 μg/mL were observed but increase in growth was seen at 5 μg/mL (Figure 4A). Similar results were obtained when PBDM1 treated MRSA was analyzed (Figure 3C). However, MRSA treated PBDM2 also showed similar effects as *S. aureus* till 10 μg/mL (98.2%) (Figure 4C). Further, PBDM1 treated VRSA showed almost 99% inhibition till 15 μg/mL concentration but lower concentrations showed increase in growth (Figure 3E). In case of VRSA treated with PBDM2 (Figure 4E) showed more than 94% inhibition till 10 μg/mL but concentration below showed no inhibition. Whereas, PBDM1 treated *E. faecalis* growth curve showed good inhibitions of 95% at 50 μg/mL but decrease in concentrations with time showed increase in growth and inhibition of more than 55% until 10 μg/mL but lower concentration of 5 μg/mL showed no inhibition as shown in Figure 3G. PBDM2 treated *E. faecalis* (Figure 4G) showed 90% inhibitions for 50, 20, and 10 μg/mL until 15 h but after that the gradual increase in growth was observed whereas, 5 μg/mL treatment showed a steady growth. For PBDM1 treated VRE, almost all of the concentrations showed inhibition of more than 52% but 50 μg/mL concentration treatment showed significant inhibition of 93% as shown in Figure 3I. Similarly, for PBDM2 treated VRE (Figure 4I) inhibition of 89% at 50 μg/mL but lower concentration until 10 μg/mL showed more 50% inhibition but 5 μg/mL showed less than 40% inhibition. Finally, for PBDM1 treated *E. coli* growth showed steady inhibition till 10 μg/mL with 96% inhibition but below this concentration no prominent inhibition was obtained (Figure 3K). Similarly, PBDM2 treated *E. coli* showed more than 92% inhibition until 10 μg/mL but concentration below had no prominent inhibition (Figure 4K). Thus, PBDM1 showed good antibacterial effects against all the tested bacteria whereas PBDM2 had similar effects toward the tested bacterial strains except in case of *E. faecalis* the antibacterial effect was moderate (50%).

Henceforth, to further validate the antibacterial activity the viability percentage assay was performed. The viability percentage of PBDM1 treated *S. aureus* was very low when treated with 50 μg/mL (5.72%) until 10 μg/mL (2.72%) but concentration below showed viability of 95% (Figure 3B). Whereas, PBDM2 treated *S. aureus* showed very low viability from 50 μg/mL (7.4%) until 10 μg/mL (2.8%) showing significant inhibitory effects, but lower concentrations showed increase in viability to 43.5% at 5 μg/mL (Figure 4B). For PBDM1 treated MRSA similar results as seen for *S. aureus* was obtained (viability less than 2.6% until 10 μg/mL) but lower concentration showed rapid increase in viability (Figure 3D). Similarly, PBDM2 treated MRSA showed no inhibition at 5 μg/mLbut concentration of 10 μg/mL and higher showed low viability around 1.8% (Figure 4D). In case of PBDM1 treated VRSA the viability was very low until 15 μg/mL (0.61%) but the concentration below (10 and 5 μg/mL) showed no prominent inhibition (Figure 3F). Whereas, PBDM2 treated VRSA showed low viability till 10 μg/mL (0.97%) but lower concentration showed prominent increase in viability up to 80% (Figure 4F). The viability of VRE and *E. faecalis* was not low in comparison to *S. aureus* and it resistant strains at lower concentrations 15and 20 μg/mL. But the viability was less than 4.6% for *E. faecalis* when treated with 50 μg/mL of PBDM1 and lower concentrations like 15 μg/mL (41.7%) and 20 μg/mL (39.5%) showed some inhibitory effects but concentration below showed no inhibition (Figure 3H). Whereas, PBDM2 treated *E. faecalis* (Figure 4H) showed overall viability less than 50% viability until 20 μg/mL but the lower concentration of 10 μg/mL showed an inhibition of less than 35.6% and concentration below showed increase in viability (63.3%). For PBDM1 treated VRE (Figures 3J, 4J) showed inhibitory effects for all the concentrations with viability below 48% and lowest viability was 6.6% at 50 μg/mL. Similarly, PBDM2 treated VRE showed viability of 10.5% at 50 μg/mL and further lower in concentration like 20 μg/mL (51.7%) to 5 μg/mL (63.9%) showed increase in viability (Figure 4J). Finally, *E. coli* (gram negative bacteria) treated with PBDM1 showed very low viability at 50 μg/mL (5.6%) and 20 μg/mL (2.9%). PBDM2 treated *E. coli* showed constant inhibition from 50 μg/mL (8.1%) to 20 μg/mL (3%). Whereas, both the PBDM peptides showed lowest viability at 10 μg/mL for PBDM1(3.5%) and PBDM2 (2.8%) but concentration below showed no prominent inhibitory effects as shown in Figures 3L, 4L, respectively. The control of the experiment was bacterial cultures without any treatment showing normal stable viability of 100%. Further, the colony forming assay was performed to confirm the antibacterial effects of PBDM peptides against *S. aureus*, MRSA, VRSA, *E. faecalis*, VRE, and *E. coli*. PBDM1 treated bacterial strains with respective MICs showed no colonies for any bacterial strains except four colonies visible for *E. faecalis* after 24 h. Similarly, in case of PBDM2 treated bacterial strains showed identical results except in case of *E. faecalis* few more number of colonies were obtained. Whereas, the negative control showed significant bacterial growth but in case of positive control (tetracycline and cefoxitin) no bacterial growth was observed (Supplementary Figure S10).

Thus, the above tests confirmed the significant antibacterial activity of PBDM peptides against *S. aureus*, MRSA, VRSA, and *E. coli*. But in case of *E. faecalis* and VRE the concentration of the peptide showing high antibacterial activity was higher in comparison to other bacterial strains. The *E. faecalis* strain used in our study was a wild type strain. We do not know if resistance to peptide could arise through plasmids, therefore we can only postulate. We have preliminary LC-MS data (unpublished) for peptide-treated VRSA protein extracts showing high levels of proteases expressed, and to our knowledge that is the only mechanism that can explain resistance in *E. faecalis*. Further studies are required to explain possible peptide resistance mechanisms. However, to further validate the antibacterial activity of PBDM peptides microscopic analysis were performed.

### Microscopic Analysis of Treated Bacterial Cells Under Phase Contrast Condition and Live/Dead Cell Imaging

**FIGURE 5:**
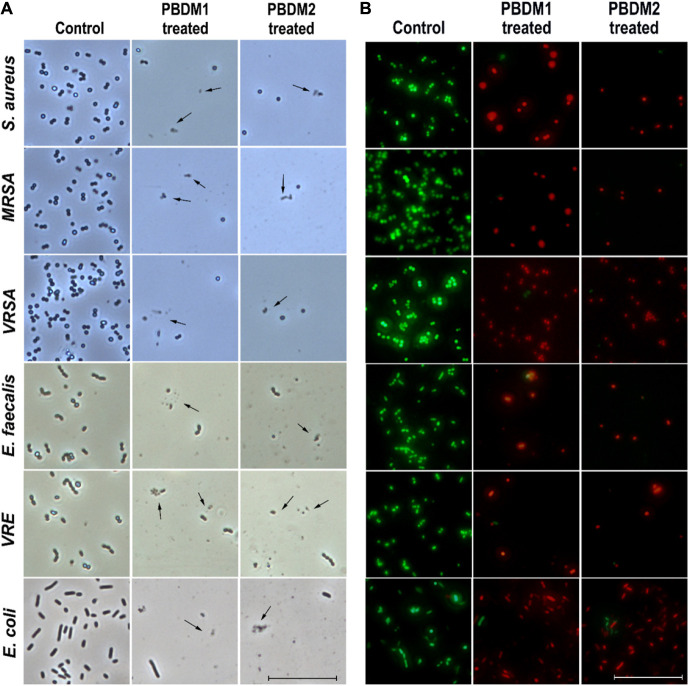
**(A)** The phase contrast microscopic image of *S. aureus*, MRSA, VRSA, *E. faecalis*, VRE, and *E. coli* after treatment with PBDM peptides, black arrows showing cell rupture with cellular debris but cells in comparison to the control. Scale is 5 μm. **(B)** The live/dead cell image of *S. aureus*, MRSA, VRSA, *E. faecalis*, VRE, and *E. coli* after treatment with PBDM peptides showing green fluorescence for live cells and red fluorescence for dead cells. Scale is 10 μm.

The phase contrast condition was used to further visually confirm the antibacterial efficacy of PBDM peptides against *S. aureus*, MRSA, VRSA, VRE, *E. faecalis*, and *E. coli*. The number of viable bacterial cells decreased after the treatment with the PBDM peptides for all the bacterial strains. The loss of cell integrity and morphological changes were observed. Further, cell wall rupture caused the cytoplasmic leakage and presence of cell debris was visualized for all the treated bacterial strains (Figure 5A). Whereas, the control bacterial cells showed high number of viable cells with no cell debris, sign of change in morphology and cellular integrity. Thus, the PBDM peptides showed prominent antibacterial effects against the bacterial samples tested.

Further, the viability of the bacterial strains in the presence of PBDM peptides were observed by the live/dead cell assay using the inverted fluorescence microscopy (Figure 5B). The bacterial samples treated with the PBDM peptides showed a visible increase in the red fluorescent dots. Thus, its clearly indicates the decrease in the viability of the bacteria (negligible green fluorescent) and increase in number of dead cells with high red fluorescent dot as compared to control samples. Due to the inhibitory effects of the PBDM peptides the number of bacterial cells were less compared to the control. Conversely, the bacterial samples without treatments were used for control, showing high numbers of live green fluorescence bacterial cells with negligible dead red fluorescence cells.

Thereafter, the ambient light illumination by optical bright field microscopy also confirmed the antibacterial effect of the PBDM peptides against *S. aureus*, MRSA, VRSA, VRE, *E. faecalis*, and *E. coli*. Decrease in the viable bacterial cell numbers and disruption of cells with loss of integrity and presence of cell debris were seen in almost all the bacterial samples in comparison to the control groups (Supplementary Figure S6). These results were in good agreement with the phase contrast analysis. Thus, the results for all the different treated bacterial samples showed prominent reduction in the viability establishing their high antibacterial potential (Stiefel et al., 2015). So, the probable mechanism of action for PBDM peptides were assumed to be via the cell wall disruption of bacterial cells. Moreover, as a lot of studies are well known for both VRE and MRSA till date. Whereas few studies are published with VRSA which was the main focus for their study. Also when we performed a thorough search in the Web of Science portal with parameter as “VRSA infection,” “*in vivo*,” “Vancomycin resistant Staphylococcus aureus” and “antibacterial activity” showed only 5 articles. However, it has been already well proven that VRSA has acquired resistance from VRE against Vancomycin. Furthermore, VRSA is also one of the pathogenic bacteria prioritized by WHO (World Health Organization) which too needs immediate check. Thus, we wanted to study the potential of the peptides against a vancomycin resistant strain. Therefore, further studies were carried out by the representative pathogenic bacterial strain, VRSA for consecutive analysis (Jelinkova et al., 2018a; Sur et al., 2020b).

### Detection of VRSA Using PBDM–5(6)–Carboxyfluorescein and Cryo-SEM Microscopic Investigation of Vancomycin Resistant Staphylococcus Aureus

**FIGURE 6:**
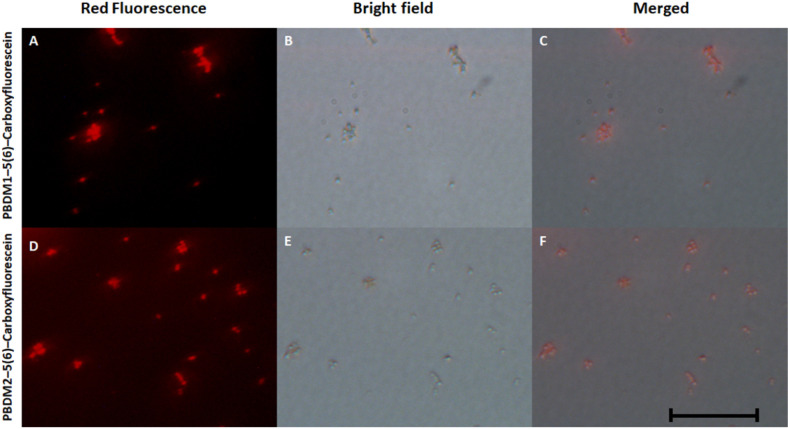
**(A,D)** showing he fluorescence microscopic image of PBDM1–5(6)–Carboxyfluorescein and PBDM–5(6)–Carboxyfluorescein interacts with the VRSA cells, respectively; **(B,C)** showing bright field image of PBDM1–5(6)–Carboxyfluorescein and PBDM2–5(6)–Carboxyfluorescein interacts with the VRSA cells; **(C,F)** showing the merged image of **(A)** with **(B)** and **(D)** with **(E)**. Scale is 20 μm.

To understand the way PBDM peptides interacts with the VRSA cells, PBDM peptides conjugates were formed, which were used for detection under fluorescence microscope. The conditions of controls were VRSA cells with and without PBDM peptides, both the control conditions showed no red fluorescence as shown in Supplementary Figure S7. Whereas the VRSA cells incubated with the conjugates showed red fluorescence emitting from the VRSA cells proving that PBDM–5(6)–Carboxyfluorescein conjugates emits a red fluorescence after interacting with the VRSA cells under fluorescence microscope. The interaction of PBDM1–5(6)–Carboxyfluorescein and PBDM2–5(6)–Carboxyfluorescein with the VRSA cells provide evidence that the cells are present in the same field showed by red fluorescence which were in identical position when observed under bright field microscope (Figure 6). Thus, it can be used to understand the interaction of peptides with the help of its conjugates emitting red fluorescence.

The analysis of VRSA with and without PBDM peptides were done using the Cryo SEM. The sample preparation by plunging method using liquid nitrogen results in the production of many layer of artifact. The best field was searched and selected for further analysis (Wu et al., 2014). The VRSA cells treated with PBDM1 showed distorted cell wall and rupture in the cell surface. These cells also showed the property to agglomerate and change in cell integrity, which is due to the after effects of cell damage by PBDM1.

**FIGURE 7:**
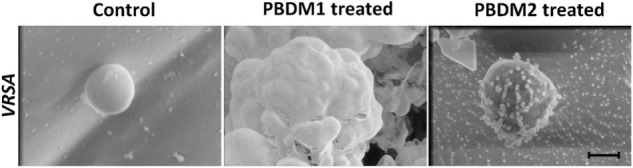
Cryo-SEM microscopic analysis of VRSA cells treated with PBDM1 and PBDM2 peptides, respectively. Control is untreated VRSA. Scale is 500 nm.

**FIGURE 8:**
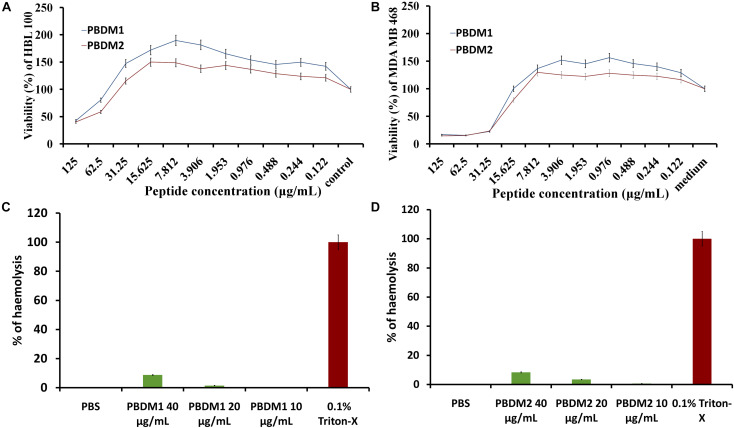
**(A,B)** are MTT assay of PBDM1 and PBDM2 against HBL100 and MDA MB 468; **(C,D)** are hemolytic assay of PBDM1 and PBDM2, where Positive control is 0.1% Triton-X and negative control PBS.

Whereas in case of VRSA without any treatment showed round spherical cell surface with intact cell membrane (Figure 8). In case of PBDM2 treated VRSA cells showed deformed globular cell surface with change in morphology but no visible rupture. Thus, the damage to VRSA cells was clearly observed due to the presence of PBDM peptides, however, the interaction was different. It can be concluded that PBDM behave like a cell penetrating peptide by destroying VRSA cell wall but in case of PBDM1 the mechanism can be the cell wall damage followed by damaged cell aggregations (Figure 7) whereas the mechanism for PBDM2 was observed to damage the VRSA cell walls forming small bubble shaped leading to change in integrity of cells and their morphology but no aggregations were observed (Figure 7). It is also likely that these bubble-shaped formations are attributed to the crystalized peptides during the Cryo-SEM procedure. Thereafter, the PBDM peptides were used to study its antibacterial effects against hospital patient bacterial samples.

### Hospital Patient Bacterial Samples in the Presence of PBDM

Probiotic Bacteriocin Derived and Modified peptides were tested on samples obtained from wounds of three different hospital patients, designated P1, P2, and P3 The samples P1A1, P1B1, and P1B2 were identified as strains of *Staphylococcus aureus*. When the samples were tested by growth curve analysis using PBDM1 showed inhibition (Supplementary Figure S2) of more than 99% for all the concentrations (50, 25, and 10 μg/mL) tested except for P1A1 (71% viability) which showed no prominent inhibition at 10 μg/mL. Whereas, PBDM2 showed inhibitory effects of 99% for all the concentrations (50, 25, and 10 μg/mL) tested. Similar, results were obtained for both the peptides when the viability percentage was performed which confirmed the significant antibacterial activity of PBDM peptides against P1 patient samples (Supplementary Figure S2A).

For samples from patient P2 were identified as *Staphylococcus epidermidis* (P2A1) and *Klebsiella pneumoniae* (P2B1, P2C1, and P2C2). The growth curve and viability percentage showed significant inhibitions of more than 98% for all the concentrations (50, 25, and 10 μg/mL) (Supplementary Figure S3A) except for sample P2A1 (72%) at 10 μg/mL (Supplementary Figure S3B). Whereas the P2 samples when tested with PBDM2 showed significant inhibition and almost negligible viability for all the concentrations tested (Supplementary Figure S3).

Lastly, the samples from patient P3 were identified as *Staphylococcus aureus* (P3A1), and *Enterobacter cloacae* (P3B1 and P3C1). PBDM1 tested against P3A1 showed prominent inhibition of 99% till 25 μg/mL (Supplementary Figure S4A) but 30% inhibition at 10 μg/mL. Whereas, PBDM1 tested against P3C1 (*Enterobacter cloacae*) was effective and caused an 98.7% inhibition till 15 μg/mL but lower concentration of 10 μg/mL showed viability of 72% (Supplementary Figures S4E,F). Growth curves in presence of PBDM1 supports the results obtained through viability percentage assay. Whereas, in case of PBDM2, all the sample from P3 patients showed significant inhibition of more than 98% for both growth curve and viability percentage assay (Supplementary Figure S4).

Thus, PBDM2 peptide showed better antibacterial activity in comparison to PBDM1 peptide even at 10 μg/mL against, P1A1, P2A1, P3A1, and P3C1 (Supplementary Table S1). So both the peptides can be used with some medicinal value against hospital associated pathogenic bacteria. PBDM peptides showed high antibacterial activity against gram positive bacteria as seen in nisin peptides but in case of VRE and *E. faecalis* the activity of PBDM peptides needed higher concentrations for prominent antibacterial efficacy. However, PBDM not only showed activity against gram positive bacteria but also was effective against gram negative bacteria which were not seen in case of nisin peptides without any modification (Jozala et al., 2015; Shin et al., 2016; Vukomanović et al., 2017; Fernández-Pérez et al., 2018). So, further *in vitro* studies using human cell lines to study toxicity and *in vivo* studies were performed to check the antibacterial activity and biocompatibility against infected balb/c mice.

### Influence and Toxicity Test of PBDM on Eukaryotic Cells

The efficiency of any novel antimicrobial agent is enhanced if the molecule is effective and non-toxic toward normal mammalian cells. Thus, cytotoxicity of PBDM peptides were evaluated using MDA MB 468 and HBL 100 cell lines by MTT assay. The HBL 100 cells showed no sign of toxicity until 31.25 μg/mL of PBDM1. However, 19% inhibition around 62.5 μg/mL with viability more than 80% was observed but higher concentrations reduces the viability (50%). Therefore, PBDM1 was found to be non-toxic at its effective MIC against HBL 100 cells but toxicity increases with concentrations higher than 62.5 μg/mL. Similarly, PBDM2 showed no toxicity until 31.25 μg/mL (MIC range) but higher concentrations showed reduction in viability (58.6 to 40%). Whereas, the MDA MB 468 adenocarcinoma cells in presence of PBDM peptides showed viability below 25% at 31.25 μg/mL and higher concentrations showed even further reduction of viability for both the peptides. At 15 μg/mL showed 79.9% viability for PBDM2 but no reduction of viability was seen for PBDM1 concentrations below showed no toxicity. The IC_50_ values of PBDM1 and PBDM2 peptides were 115.02 and 103.8 μg/mL for HBL 100 and 67.55, and 61.52 μg/mL for MDA MB 468, respectively (Supplementary Table S2). However, the MIC value of the peptides were much lower than the IC_50_ values of the peptides obtained. Thus, PBDM peptides were effective against the cancer cells and pathogenic bacterial cells but showed no prominent toxicity against normal human epithelial cells at the MICs range as shown in Figures 8A,B.

Finally, the hemolytic effects of the PBDM peptides showed negligible hemolysis that is less than 9% at 40 μg/mL and lower concentration showed less than 4% in case of PBDM2 (20 μg/mL) and less than 1.5% for PBDM1 (20 μg/mL) which is very close to negative control (PBS) as shown in Figures 8C,D. But peptides like nisin have hemolysis at 1000-fold higher than its antimicrobial activity concentration (Jozala et al., 2015). Usually peptides have higher percentage of hemolysis even in low concentration which makes them complicated for the actual application of medicine (Sanches et al., 2015). Thus, the results showed that the PBDM peptides without any modification has a strong antimicrobial effects with no prominent toxicity and with good hemocompatibility toward mammalian cells, *in vitro* (Jelinkova et al., 2018a). Further, *in vivo* analysis of the PBDM peptides using balb/c mice were performed.

### *In vivo* Analysis of Infected Balb/c Treated With PBDM Peptides

**FIGURE 9:**
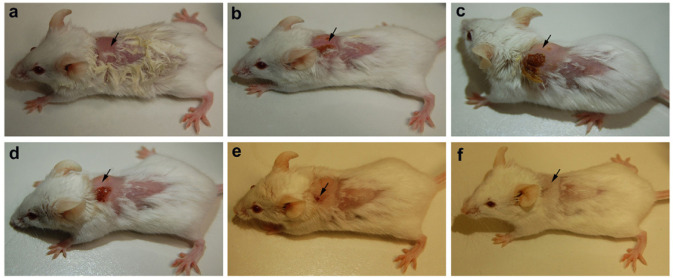
Balb/c mice infected with VRSA and treated with PBDM1. **(A)** is Day 1, **(B)** is Day 5, **(C)** is Day 11, **(D)** is Day 12, **(E)** is Day 17, and **(F)** is Day 19.

**FIGURE 10:**
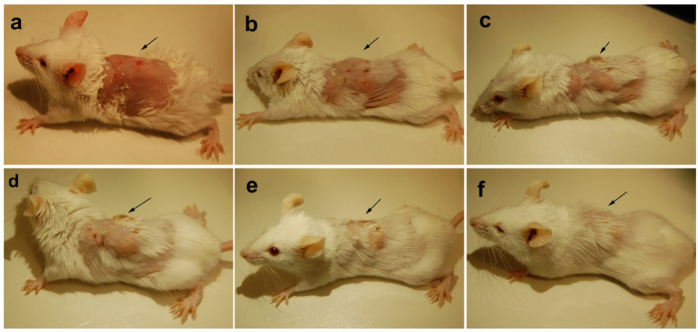
Balb/c mice infected with VRSA and treated with PBDM2. **(A)** is Day 1, **(B)** is Day 4, **(C)** is Day 10, **(D)** is Day 14, **(E)** is Day 17, and **(F)** is Day 23.

The animals were infected with VRSA by subcutaneous injection. After the initiation of the infection and its spreading in the near neck dermal region of the mouse, it led to wound and swells formation. Henceforth, after the wound and swelling was prominent as shown in Figures 9, 10, the treatment with the PBDM peptides was started. With the initiation of treatment day by day the healing of the wound and reduction in infection showed the recovery of the mice from the severe infection state to normal healthy state as shown in Figures 9, 10, respectively. The PBDM1 treated infected animals showed faster recovery from the infection when compared to PBDM2 treated infected animals. Thus, with the commence of VRSA infection in mice and the start of treatment with the concentration of 10 μg/mL of PBDM1 and 15 μg/mL of PBDM2 peptides, showed complete cure of the mice from infection. One of the prime findings was the complete cure of the infection in mice by combination of topical administration and subcutaneously injecting the PBDM peptides.

The infected untreated control (Set two) was prepared by the introduction of VRSA infection and was monitored (Supplementary Figure S8). The infected untreated control set of animals were monitored and under experimental condition showed the morbidity were 100% within 14 days, wherein internal infection growth along with the dermal infection growth was observed. The untreated infected mice underwent dermal infection alongside internal growing infection causing fever and whole body twitching (Supplementary Figure S9). Whereas, the uninfected control animal group was maintained and monitored throughout the experiment and showed no changes in its condition (Supplementary Figure S8).

Thereafter the mice treated with PBDM peptides were being further monitored and kept under observation for one more week after recovery and they were alive, normal and healthy with no unusual behavior. Finally, PBDM1 and PBDM2 peptides were able to treat VRSA infection showing no negligible toxicity toward the balb/c mice and they were biocompatible showing full recovery after treatment. Another major finding was PBDM1 was more effective in short number of days compared to PBDM2 but the concentration needed for PBDM1 was higher than PBDM2. Overall, MTT assays have showed that PBDM2 was more toxic than PBDM1, likely due to the presence of two extra positively charged amino acids (Arg5 and Lys10). From peptide structure point of view, both peptides form a U-shape 3D structure, however, PBDM1 forms a more stable structure due to beta bridge between Lys2 and Leu11 residues. We hypothesize that the rigidity of PBDM1 structure may also contribute to poor toxicity as compared to PBDM2 structure which has less rigid U-shape 3D structure (due to repulsion between positively charged lysines). Increased positive charge has been previously reported in antimicrobial peptides (reference). We can also hypothesize that the bioavailability of the peptides was highly influenced by the concentration used, and hence the effect on recovery time. Electron microscopy images have shown different effects for peptides on bacterial cell morphology. PBDM1 treatment showed cellular shrinkage caused by rupture of the cell walls and leakage of cytoplasm. On the other hand, PBDM2 treatment resulted in bubble shaped debris on the bacterial cell wall. Thus, PBDM peptide can further be used for clinical studies to develop theranostics against bacterial infection as an alternative to antibiotics.

## Conclusion

The outcome of the whole experiment helped us to ensue new antibacterial peptides to overcome the limitation of antibiotics to treat the multidrug resistant pathogenic bacterial infections. The designing and synthesis of PBDM–5(6)–Carboxyfluorescein conjugates (purification by HPLC-UV) allowed real-time follow-up of peptide effect. The *in silico* structural analysis of the PBDM peptides correlated with biophysical and spectroscopic characterization that was performed by MALD-TOF MS, ATR-FTIR, absorbance and fluorescence emission spectra. The PBDM peptides were used against *S. aureus*, MRSA, VRSA, VRE, *E. faecalis*, and *E. coli* to study the MICs, growth curves, viability and CFUs of the bacteria in their presence, which proves that they have a significant antibacterial effects against the pathogenic bacterial strains studied. Further, the activity of the PBDM was proven by testing it against the pathogenic live hospital samples (variants of *Staphylococcus aureus*, *Enterobacter cloacae*, *Klebsiella pneumonia*e, and *Staphylococcus epidermidis*). Further, to confirm the antibacterial activity visually, the phase contrast and bright field microscopic images analysis were performed, which revealed that PBDM peptides are effective against bacterial cells causing loss of cell integrity, change in morphology and cell disruption leading to cell leakage and presence cell debris. The live/dead cell imaging of PBDM treated bacterial strains supported the antibacterial activity. Thereafter, Cryo-SEM imaging revealed the mechanism of the PBDM1 as it was found to damage the cell wall causing aggregations of other VRSA cells and PBDM2 interacts with the cell wall causing bubble like structure (suspected to be crystalized peptides) and change in cell integrity and morphology but no cell wall damage was observed.

Finally, the *in vitro* toxicity test was done for PBDM using human blood cells, mammary gland epithelial cells (HBL 100) and mammary gland adenocarcinoma cells (MDA MB 468)proving it has some effects on cancer cells but negligible toxicity against normal mammary gland epithelial cells at similar concentrations with negligible hemolysis. IC_50_ values in comparison to the MIC value shows the effective concentration for antibacterial activity was lower than the IC_50_ values. Lastly, the *in vivo* analysis of the VRSA infected balb/c mice treated with PBDM1 and PBDM2 showed the recovery from severe to healthy condition showing with negligible toxicity and high biocompatibility. PBDM1 was more effective in less number of days than PBDM2. Thus, PBDM peptides can be considered a great replacement to antibiotics as it helps to overcome the limitations exhibited by most of the antibiotics.

## Data Availability Statement

The original contributions presented in the study are publicly available. This data can be found in GenBank, under accession numbers MT792519–MT792528.

## Ethics Statement

The studies involving human participants were reviewed and approved by the Ethics Committee of Trauma Hospital in Brno, Czechia in accordance to Act No. 378/2007 coll. The patients/participants provided their written informed consent to participate in this study. The animal study was reviewed and approved by the Ethics Commission at the Faculty of AgriSciences, Mendel University in Brno, Czechia in accordance with Act No. 246/1992 Coll.

## Author Contributions

All the authors contributed researched data, wrote their respective sections in the manuscript, reviewed and edited the article substantially. AMa performed most of the experiments including designing peptide, spectrophotometry, microbiological tests, microscopy, some part of animal experiment, and wrote the most part of the manuscript. YH helped in the MD simulation and development of the mechanism of action. VS helped in animal experiment. VM helped in the synthesis of the peptide and the characterization of the peptide. SB helped in microbiological test. HM and RG helped with different experiments. RV provided the hospital samples. AMo supervised the whole experiment.

## Conflict of Interest

The authors declare that the research was conducted in the absence of any commercial or financial relationships that could be construed as a potential conflict of interest.

## Footnotes

1 blast.ncbi.nlm.nih.gov/Blast.cgi

2 https://www.ebi.ac.uk/Tools/msa/clustalo/

## Supplementary Material

FIGURE S1 Multiple Sequence alignment of the four peptides.

FIGURE S2 Growth curve and viability percentage of bacterial samples from P1.

FIGURE S3 Growth curve and viability percentage of bacterial samples from P2.

FIGURE S4 Growth curve and viability percentage of bacterial samples from P3.

FIGURE S5 The phylogenetic tree of the hospital sample from P1, P2, and P3 patients.

FIGURE S6 The bright field microscopic images.

FIGURE S7 The bright field and fluorescence microscopic image of control VRSA cells with and without peptide and no presence of dye.

FIGURE S8 The uninfected control balb/c without treatment and infection.

FIGURE S9 The Infected untreated control with infection by VRSA and no treatment.

FIGURE S10 The Colony Forming Unit assay.

FIGURE S11 Characterization of peptides.

TABLE S1 Overview of the antibacterial activity of PBDM peptide against Hospital samples from P1, P2, and P3.
