# Supplementary material for: Characterization and in vitro Analysis of Probiotic-Derived Peptides Against Multi Drug Resistance Bacterial Infections
Source: Front Microbiol. 2020 Aug 25;11:1963. doi: 10.3389/fmicb.2020.01963 (PMC7477325; doi:10.3389/fmicb.2020.01963)
Supplement: FIGURE S1 — Multiple Sequence alignment of the four peptides. [file Data_Sheet_1.pdf]

Supplementary Data for:

Characterization and *in vitro* analysis of probiotic-derived peptides against drug resistance bacterial infections

Aninda Mazumdar[1,2*], Yazan Haddad[1,2], Vishma Pratap Sur[1,2], Vedran Milosavljevic[1,2], Sukanya Bhowmick[1,2], Hana Michalkova[1], Roman Guran[1,2], Radek Vesely[3], Amitava Moulick[1,2*]

[1]*Department of Chemistry and Biochemistry, Mendel University in Brno, Zemedelska 1, CZ-613 00 Brno, Czech Republic*

[2]*Central European Institute of Technology, Brno University of Technology, Purkynova 123, CZ-612 00 Brno, Czech Republic*

[3]*Department of Traumatology at the Medical Faculty, Masaryk University and Trauma Hospital of Brno, Ponavka, Czech Republic*

***Corresponding author**

Aninda Mazumdar, Department of Chemistry and Biochemistry, Mendel University in Brno, Zemedelska 1, CZ-613 00 Brno, Czech Republic; E-mail: anindamazumdar@gmail.com, xmazumda@mendelu.cz; phone: +420545133350

Amitava Moulick, Department of Chemistry and Biochemistry, Mendel University in Brno, Zemedelska 1, CZ-613 00 Brno, Czech Republic; E-mail: amitavamoulick@gmail.com; phone: +420545133350

1. Peptide designing

```
m2386       DSIRDVSPTFNKIRRWFDGLFK---    22
m2163       KRKCPKTPFDNTPGAWFAHLILGC-    24
PBDM1       -------------YKWFAHLIKGLC    12
PBDM2       -------------YKWFRHLIKKLC    12
                           **  *:
```

Figure S1: Multiple Sequence alignment of the four peptides.

2. Growth curve and viability percentage of the hospital samples

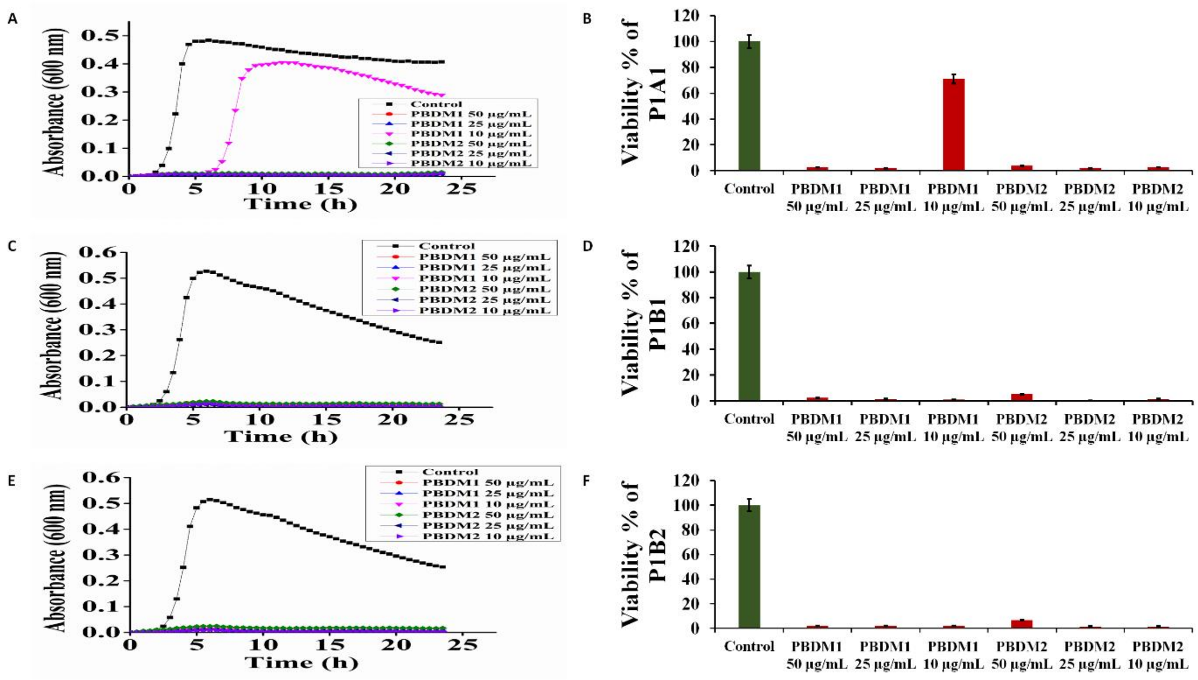

Figure S2: Growth curve and viabilty percentage of the bacterial samples obtained from patient P1 after treatment with PBDM peptides. Data represent the mean ±SD, n=3.

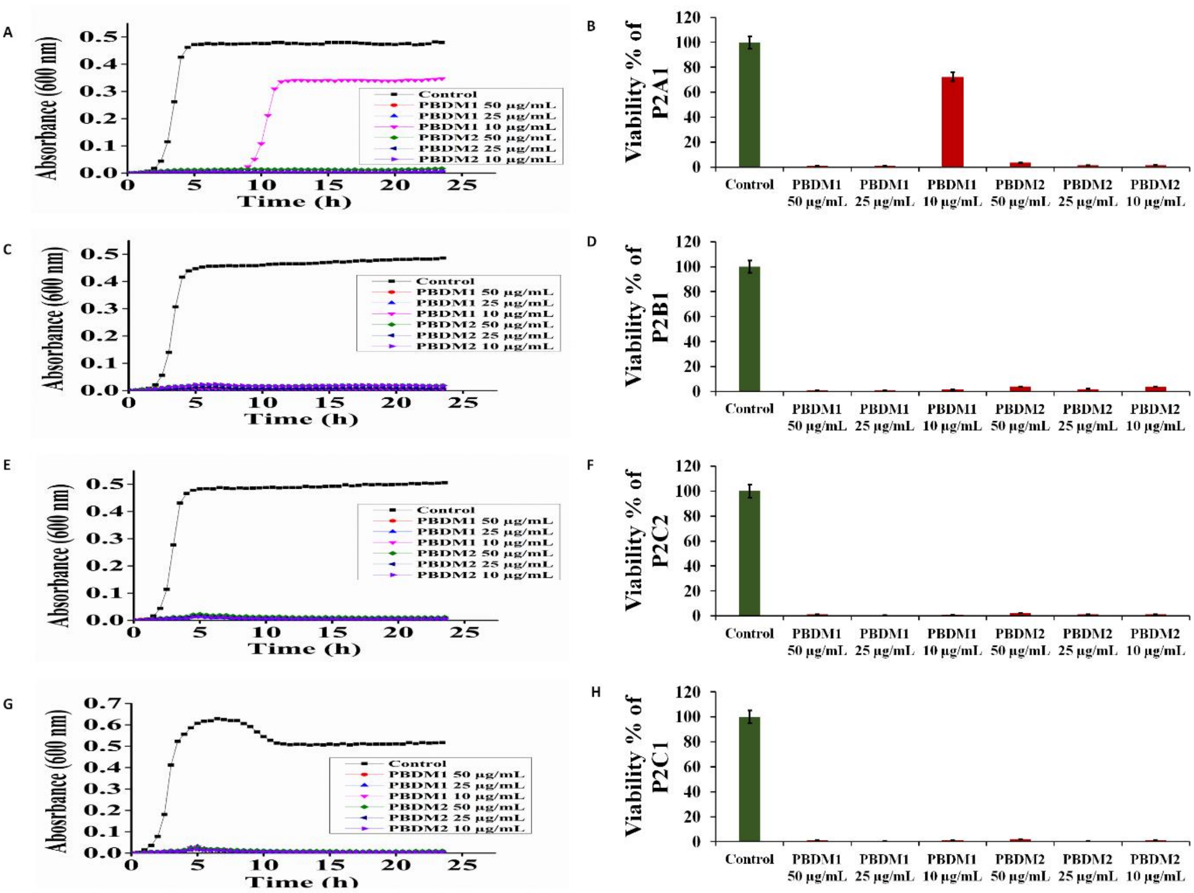

Figure S3: Growth curve and viabilty percentage of the bacterial samples obtained from patient P2 after treatment with PBDM peptides. Data represent the mean ±SD, n=3.

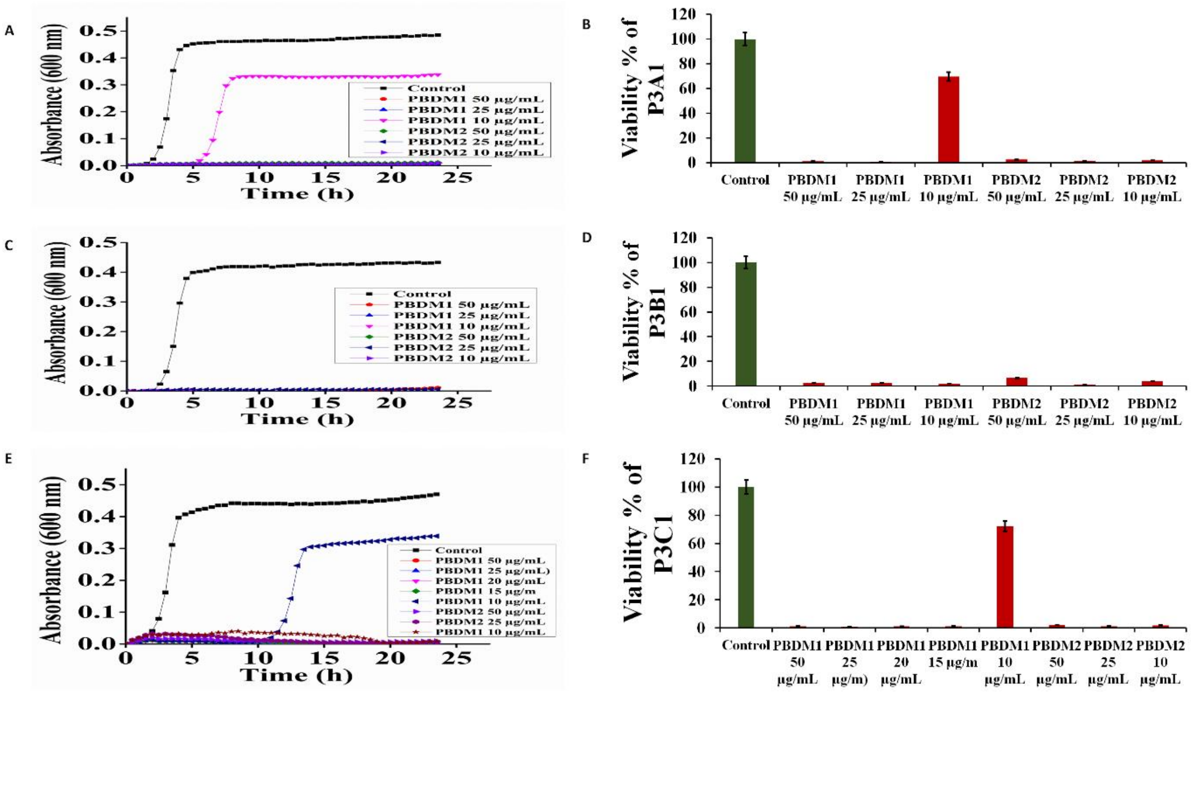

Figure S4: Growth curve and viabilty percentage of the bacterial samples obtained from patient P3 after treatment with PBDM peptides. Data represent the mean ±SD, n=3.

Table S1: Overview of the antibacterial activity of PBDM peptide against Hospital samples from P1, P2 and P3.

Sample names	*Samples obtained and specific name*	*Sample identified*	*PBDM1 MIC concentration (µg/mL)*	*PBDM2 MIC concentration (µg/mL)*
P1	P1A1	*Staphylococcus aureus*	25	10
	P1B1	*Staphylococcus aureus*	10	10
	P1B2	*Staphylococcus aureus*	10	10
P2	P2A1	*Staphylococcus epidermidis*	25	10
	P2B1	*Klebsiella pneumoniae*	10	10
	P2C1	*Klebsiella pneumoniae*	10	10
	P2C2	*Klebsiella pneumoniae*	10	10
P3	P3A1	*Staphylococcus epidermidis*	25	10
	P3B1	*Enterobacter cloacae*	10	10
	P3C1	*Enterobacter cloacae*	15	10

3. Multiple sequence alignment by clustal omega

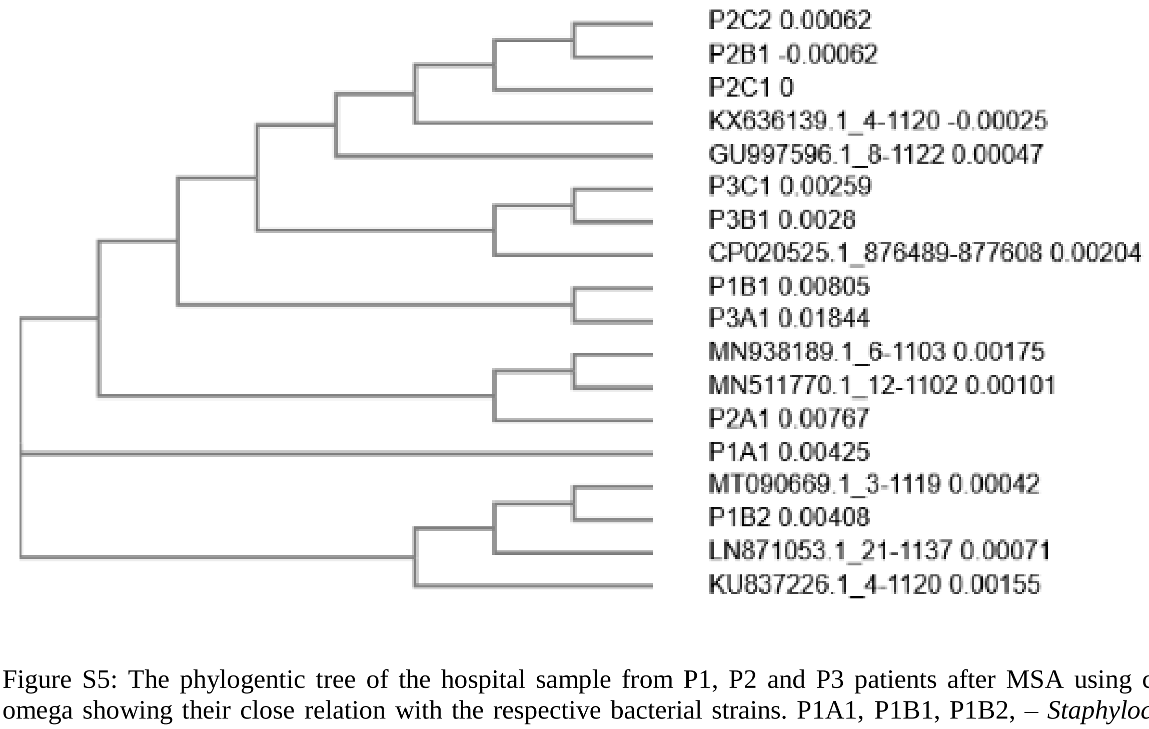

Figure S5: The phylogentic tree of the hospital sample from P1, P2 and P3 patients after MSA using clustal omega showing their close relation with the respective bacterial strains. P1A1, P1B1, P1B2, – *Staphylococcus aureus;* P2A1, P3A1 – *Staphylococcus epidermidis*; P2B1, P2C1, P2C2 – *Klebsiella pneumoniae*; P3B1 and P3C1 – *Enterobacter cloacae*.

4. *Bright feild micropscopic analyis of bacterial cells treated with PBDM peptides*

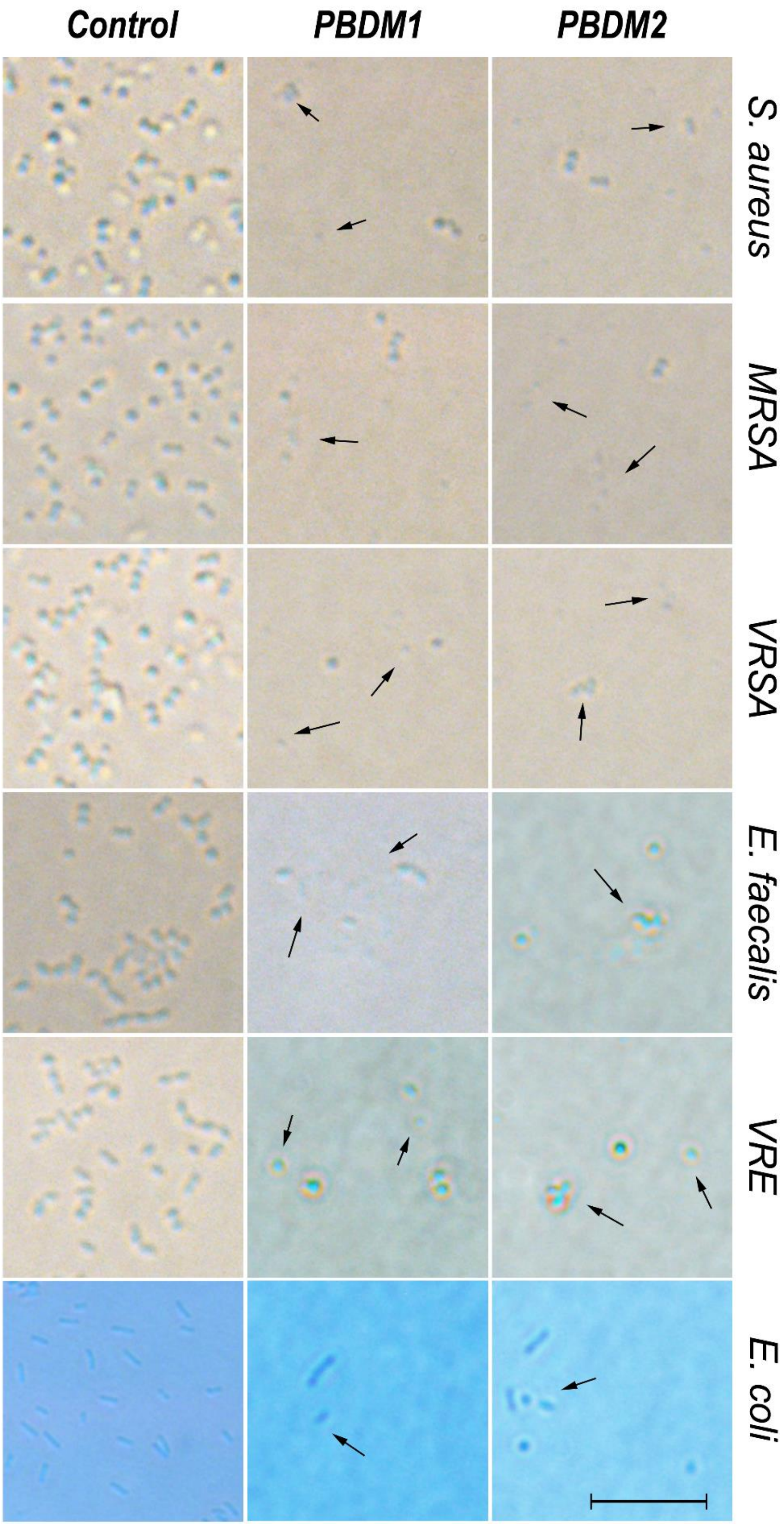

Figure S6: The bright field microscopic images of *S. aureus*, MRSA,VRSA, *E. faecalis*, VRE and *E. coli* after treatment with PBDM peptides, black arrows showing cell rupture, change in cellular morphoology and cell debris but cells in the control were in healthy condition. Scale is 10 µm.

5. ***Calculation of the hemolysis***

The percentage of hemolysis was calculated according to the following equation:

$$h = \frac{A_t - A_c}{A_{100\%} - A_c} \times 100 \, ,$$

Where, h is the percentage of hemolysis; A_c is the absorbance of the supernatant from negative control (PBS, pH 7.4); A_t is the absorbance of the supernatant from the samples incubated with the AL3; and $A_{100\%}$ is the absorbance of the supernatant of positive control (0.1% Triton X-100), which causes complete lysis of RBCs

6. ***Detection of VRSA using PBDM–5(6)–Carboxyfluorescein***

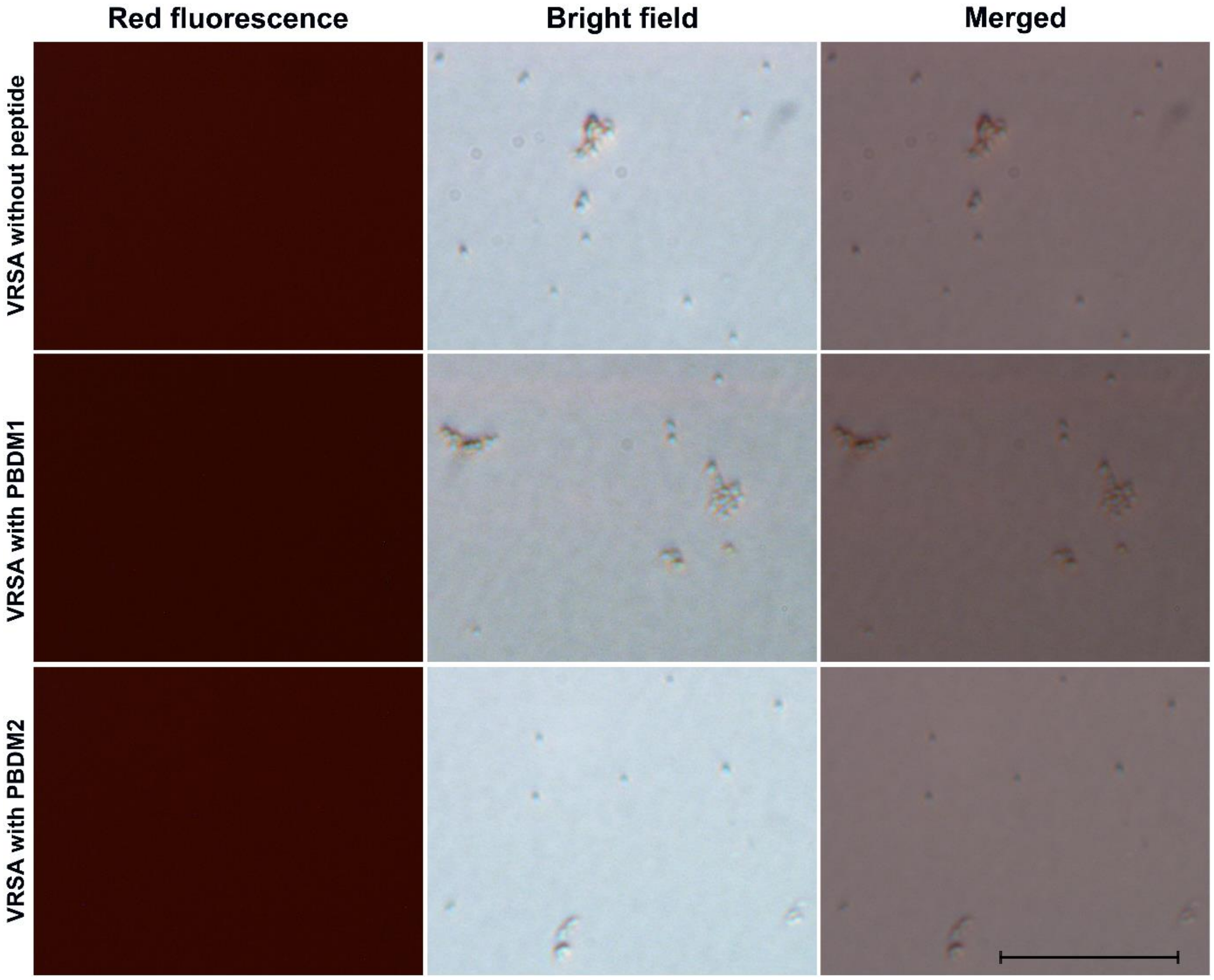

Figure S7: The bright feild and fluorescence microscopic image of control VRSA cells with and without peptide and no presence of dye. Scale is 20 µm.

7. *In vivo of PBDM peptide*

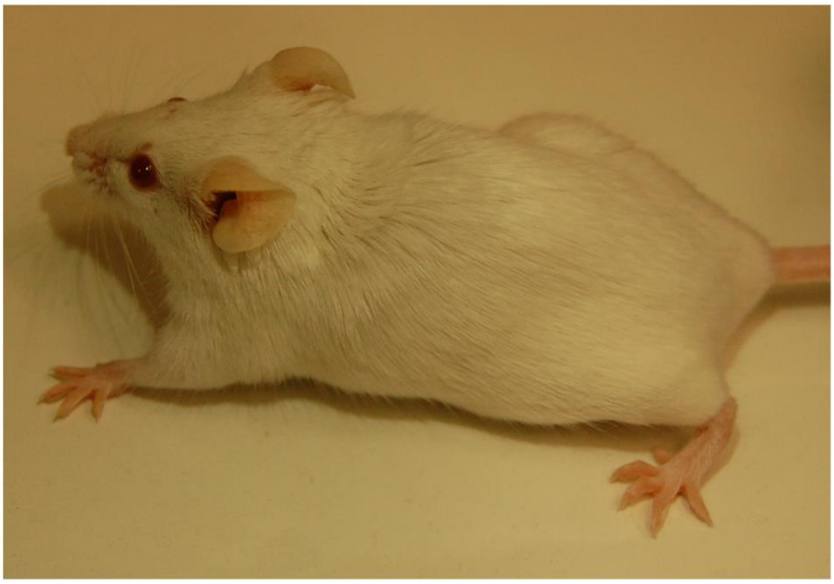

Figure S8: The uninfected control balb/c without treatment and infection.

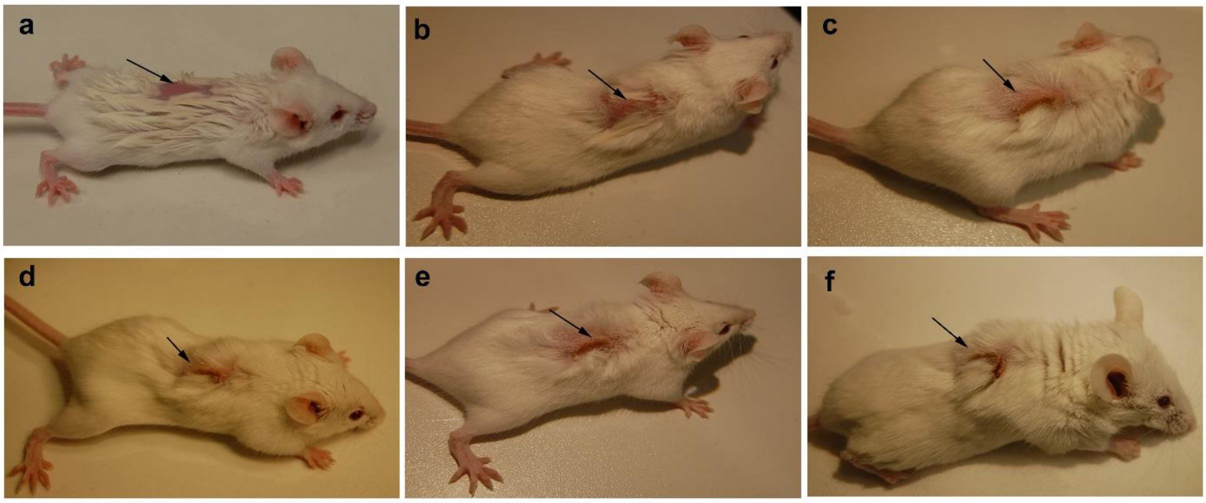

Figure S9: The Infected untreated control with infection by VRSA and no treatment. a is Day 1, b is Day 3, c is Day 6, d is Day 7, e is Day 8, and f is Day 14.

8. *Purification of peptides by HPLC-UV*

In HPLC-UV, the column was used Kinetex EVO C18 (150 x 4.6 mm, 5 µm). Wavelength was set to 214 nm. Mobile phase A consisted of water + 0.1% formic acid. Mobile phase B consisted of methanol + 0.1% formic acid. Gradient mode: 0 min 3% B –> 30 min 50% B –> 50 min 3% B –> 50.1 min 85% B –> 55 min 85% B –> 55.1 min 3% B –> 60 min 3% B –> STOP. Flow rate was 0.5 mL/min. Injected sample volume was 20 µL. Prior analyses the samples were diluted 100x with water + 0.1% formic acid.

9. Colony Forming Unit (CFU) assay

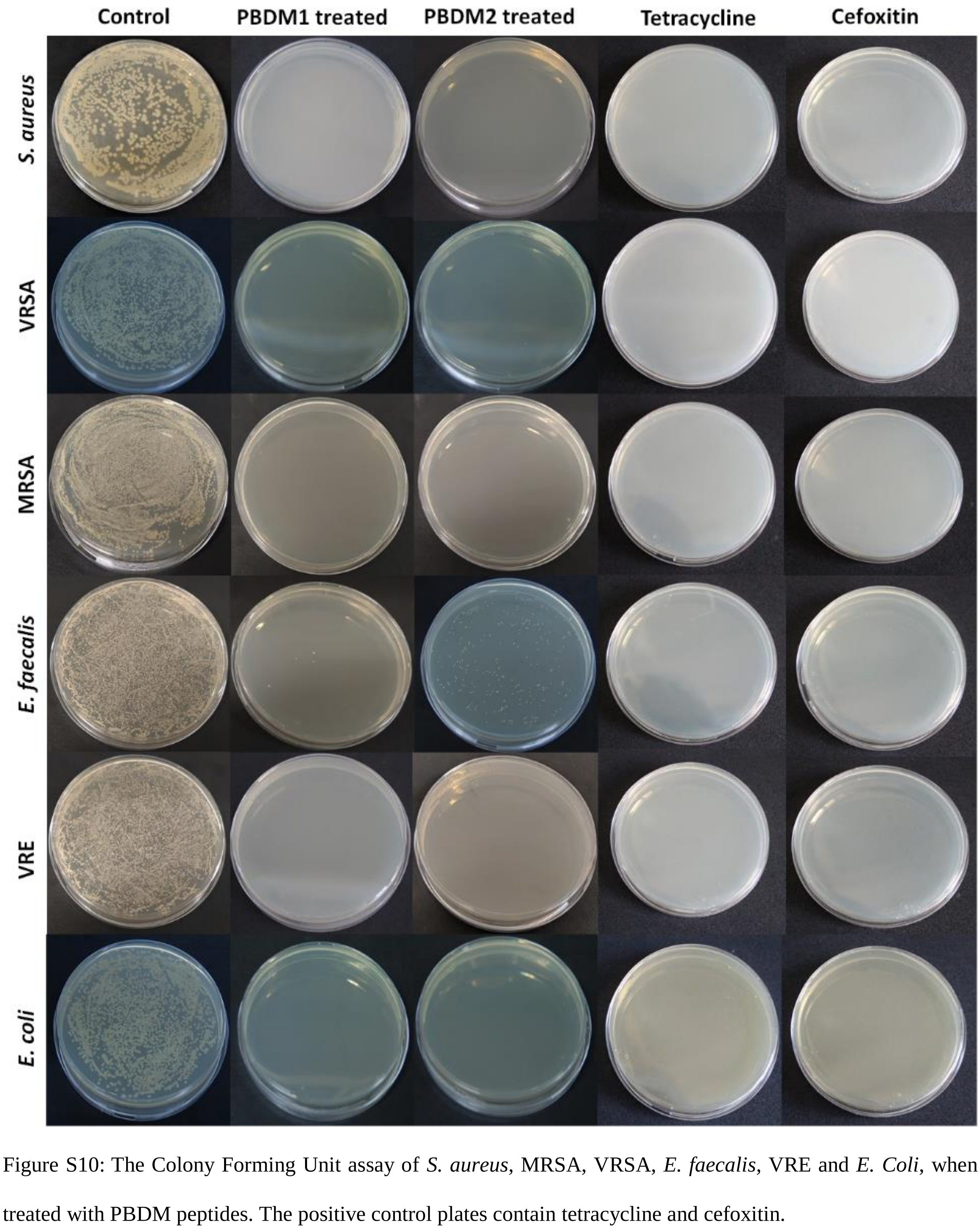

Figure S10: The Colony Forming Unit assay of *S. aureus*, MRSA, VRSA, *E. faecalis*, VRE and *E. Coli*, when treated with PBDM peptides. The positive control plates contain tetracycline and cefoxitin.

10. Cytotoxicity of the PBDM peptides defined as IC_{50}

The IC_{50} was the concentration of the peptides required for 50% inhibition of the growth of the HBl 100 and MDA MB 468 cells. The formula used was:

$$y = ax + b;$$

Where y is the percentage of the inhibition, a is the slope of the graph, x is the concentration of peptides and b is the y intercept.

Table S2: IC50 values of PBDM peptides.

Name of the cell lines	*IC_{50} of PBDM1 (µg/mL)*	*IC_{50} of PBDM2 (µg/mL)*
HBL 100	115.02	103.8
MDA MB 468	67.55	61.52

11. Characterization of PBDM

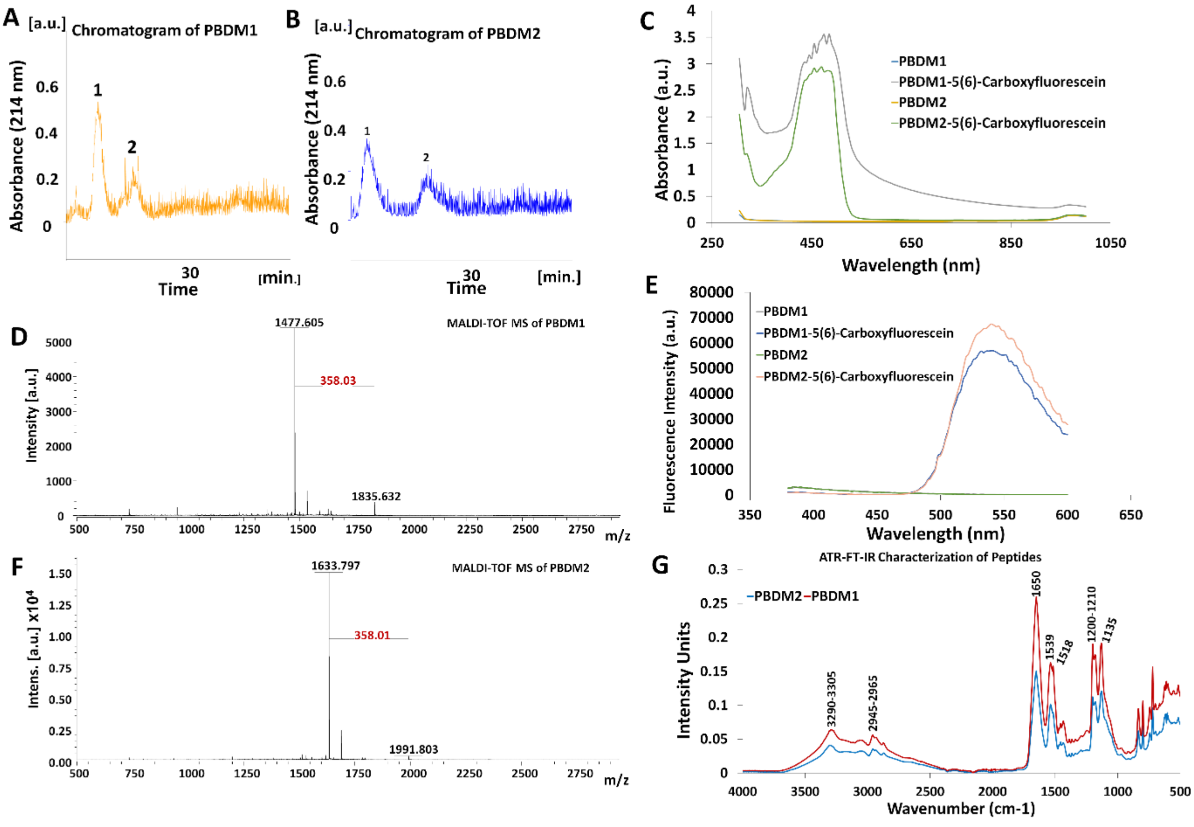

Figure S11: Characterization of peptides A) HPLC-UV chromatogram of PBDM1 peptide (Peak1) and its conjugate (Peak2), B) HPLC-UV chromatogram of PBDM2 peptide (Peak1) and its conjugate (Peak2); HPLC system consisted of two pumps ESA Model 584 and an autosampler ESA Model 542 (ESA Inc., Chelmsford, USA). C) The Absorbance spectra of PBDM and its conjugates. D) MALDI-TOF mass spectrum of peptide

PBDM1 and its conjugate with 5(6)-Carboxyfluorescein N-hydroxysuccinimide ester (473.39 Da). The mass difference of 358.03 belongs to 5(6)-Carboxyfluorescein without molecule of N-hydroxysuccinimide (115.09 Da), as it was detached after conjugation with peptide; E) The fluorescence spectra of PBDM and its conjugates; F) MALDI-TOF mass spectrum of PBDM2 and its conjugate, similar as PBDM1; G) ATR-FT-IR analysis. Characteristic amide I band at 1650 cm^{-1} is indicative of random coil secondary structure of peptides.
